# A Network Meta-Analysis of Efficacy and Evaluation of Safety of Subcutaneous Pegylated Interferon Beta-1a versus Other Injectable Therapies for the Treatment of Relapsing-Remitting Multiple Sclerosis

**DOI:** 10.1371/journal.pone.0127960

**Published:** 2015-06-03

**Authors:** Keith Tolley, Michael Hutchinson, Xiaojun You, Ping Wang, Bjoern Sperling, Ankush Taneja, Mohammed Kashif Siddiqui, Elizabeth Kinter

**Affiliations:** 1 Tolley Health Economics Ltd., Buxton, United Kingdom; 2 St. Vincent’s University Hospital, Dublin, Ireland; 3 Biogen Idec Inc., Cambridge, MA, United States of America; 4 HERON Commercialization—A Parexel Company, Chandigarh, India; Groningen Research Institute of Pharmacy, NETHERLANDS

## Abstract

Subcutaneous pegylated interferon beta-1a (peginterferon beta-1a [PEG-IFN]) 125 μg every two or four weeks has been studied in relapsing-remitting multiple sclerosis (RRMS) patients in the pivotal Phase 3 ADVANCE trial. In the absence of direct comparative evidence, a network meta-analysis (NMA) was conducted to provide an indirect assessment of the relative efficacy, safety, and tolerability of PEG-IFN versus other injectable RRMS therapies. Systematic searches were conducted in MEDLINE, Embase, and the Cochrane Library, and conference proceedings from relevant annual symposia were hand-searched. Included studies were randomized controlled trials evaluating ≥1 first-line treatments including interferon beta-1a 30, 44, and 22 μg, interferon beta-1b, and glatiramer acetate in patients with RRMS. Studies were included based on a pre-specified protocol and extracted by a team of independent reviewers and information scientists, utilizing criteria from NICE and IQWiG. In line with ADVANCE findings, NMA results support that PEG-IFN every 2 weeks significantly reduced annualized relapse rate, and 3- and 6-month confirmed disability progression (CDP) versus placebo. There was numerical trend favoring PEG-IFN every 2 weeks versus other IFNs assessed for annualized relapse rate, and versus all other injectables for 3- and 6-month CDP (6-month CDP was significantly reduced versus IFN beta-1a 30 μg). The safety and tolerability profile of PEG-IFN beta-1a 125 μg every 2 weeks was consistent with that of other evaluated treatments. Study limitations for the NMA include variant definitions of relapse and other systematic differences across trials, assumptions that populations were sufficiently similar, and inability to perform NMA of adverse events. With similar efficacy compared to other RRMS treatments in terms of annualized relapse rate and 3- and 6-month CDP, a promising safety profile, and up to 93% reduction in number of injections (which may improve adherence), PEG-IFN every 2 weeks offers a valuable alternative treatment option for patients with RRMS.

## Introduction

Multiple sclerosis (MS) is a chronic, neuro-inflammatory and neurodegenerative autoimmune disorder affecting the central nervous system (CNS) characterized clinically by recurring episodes of neurological symptoms and increasing disability over time. More than 2.1 million people are affected by MS worldwide with approximately 400,000 cases reported in the United States (US) and 600,000 cases in the European Union [1]. About 85% of patients present with relapsing forms of MS, while the other 15% present with steady progressive disability (primary progressive MS) [2,3].

Current management of relapsing-remitting MS (RRMS) involves the use of disease-modifying therapies (DMTs), which affect the course of the disease by suppressing the autoimmune response [4,5]. DMTs aim to reduce the frequency of relapses as well as slow disease progression and disability accumulation [6,7]. In a data analysis of patients with RRMS designated to the placebo arms of clinical trials, 30% of all relapses led to a confirmed Expanded Disability Status Scale (EDSS) score increase of ≥1.0. The high percentage of patients who experience permanent disabilities following relapse highlights the need for treatments that reduce sustained disability progression [8].

There are several injectable DMTs and oral DMTs approved for the treatment of RRMS. Recommended first-line therapies include interferons (IFNs [IFN beta-1a and IFN beta-1b]) and glatiramer acetate (GA). IFN and GA are injectables that range in dosing frequency from once daily (OD; i.e. GA) to once weekly (QW; i.e. IFN beta-1a) [4,9,10]. To date, clinical studies have demonstrated IFN beta-1a as achieving a reduction in both the frequency of relapses and the risk of confirmed disability progression (CDP), while IFN beta-1b and GA have only demonstrated significant reductions in the frequency of relapses [11]. With efficacy and safety data spanning more than 20 years, IFNs and GA have been regarded as the foundation of MS therapy [12]. However, attainment of optimal effects from these treatments in clinical practice has been limited by drug instability and their short half-life, IFN neutralizing antibodies (NAbs) that reduce efficacy [13–20], and poor treatment compliance due to the frequency of IFN/GA dosing schedules [21]. In a database study of 6,680 MS patients, adherence to IFNs and GA was evaluated via medication possession ratio. Overall, patients treated with QW injections had significantly higher odds of adherence than patients treated with medications requiring three to seven times weekly injections [22]. Similarly, findings from the Global Adherence Project, a multicenter real-world observational study of adherence to DMTs in patients with RRMS, also revealed that adherence is inversely related to the frequency of injection. Patients adherent to treatment reported significantly better quality of life and fewer cognitive issues compared to their non-adherent counterparts [23]. Therefore, there remains a clinical need for first-line treatment interventions with similar risk-benefit profiles (i.e. which are at least as efficacious and safe) as currently available IFNs based on randomized controlled trial (RCT) evidence, but with less burdensome administration. Reducing injection frequency can improve adherence and result in increased efficacy in terms of fewer relapses, reduced risk of disease progression, and reduced costs.

Pegylated IFN beta-1a (peginterferon beta-1a), administered subcutaneously at a dosing of 125 μg every 2 weeks, was approved in 2014 for the treatment of relapsing MS (US regulatory approval) and RRMS (EU regulatory approval), with a lower injection frequency than other approved injectable DMTs. The efficacy and safety of peginterferon beta-1a in the treatment of RRMS has been demonstrated in the ADVANCE trial, a Phase 3, randomized, double-blind, 2-year study with a placebo-controlled first phase. At Week 48 of the ADVANCE trial (end of Year 1), compared with placebo, peginterferon beta-1a every 2 weeks demonstrated a statistically significant reduction in annualized relapse rate (ARR; rate ratio [RR]: 0.644, 95% CI 0.500–0.831, *P* = 0.0007 [24]), 3-month CDP (CDP3M; hazard ratio [HR]: 0.62, 95% CI 0.40–0.97, *P* = 0.0383 [24]), and 6-month CDP (CDP6MHR: 0.46, 95% CI 0.26–0.81, *P* = 0.0069, [25]). Multiple comparative efficacy studies for the well-established injectable therapies have been previously published [26–28]; however, to date, no RCTs comparing peginterferon beta-1a with other injectable DMTs have been conducted. This study was therefore designed to provide a comparison of the recently approved peginterferon beta-1a with other existing injectable therapies for RRMS, and to provide data for evidence-based health technology assessment and decision making.

A network meta analysis (NMA) is a method for synthesizing available direct and indirect evidence identified through a systematic literature review. By linking interventions of interest through a common reference comparator it is possible to compare the efficacy and safety of alternative therapies when there is limited or no head-to-head evidence available [29,30]. NMAs can therefore be used to inform clinical decision making, support country specific reimbursement decisions, as well as to provide clinical efficacy data for economic evaluations.

The objectives of this study were to conduct a systematic review and NMA to evaluate the relative efficacy and safety of peginterferon beta-1a compared to other injectable DMTs approved for the treatment of RRMS.

## Methods

### Systematic Literature Review

A comprehensive systematic literature review was conducted based on a pre-specified protocol [31]. Using a combination of medical subject headings (MeSH) and free-text terms for specified interventions in MS, searches were conducted for relevant articles indexed in MEDLINE, Embase, and the Cochrane Library databases. Searches were conducted in March 2014 with no temporal limits and articles were limited to those published in English.

Proceedings of scientific meetings were searched for potentially relevant abstracts for the period of 2009 through 2013. The meetings included were the American Academy of Neurology, American Neurological Association, Americas Committee for Treatment and Research in Multiple Sclerosis, European Committee for Treatment and Research in Multiple Sclerosis, and European Federation of Neurological Societies. To ensure all relevant literature was included in the review, supplementary searches were conducted in trial registries (ClinicalTrials.gov and the *meta*Register of Controlled Trials). Search details are provided in the Supplementary Appendix (Table A in S1 Appendix). The published clinical trial results for peginterferon beta-1a were supplemented with additional 2-year ADVANCE study safety data (Kieseier et al., 2014 [32], data on file; all safety input data can be provided upon request) to allow for comparison across safety outcomes [24,25].

### Selection of Studies and Data Abstraction

Titles and abstracts of citations returned from the electronic database, conference, and registry searches were initially reviewed for eligibility by applying a pre-defined set of inclusion/exclusion criteria to each citation. These same criteria were re-applied to the full publications by two independent reviewers, and any discrepancies between reviewers were reconciled by a third independent reviewer.

The population of interest included patients with RRMS or a patient population with a subgroup composed of ≥80% of patients with RRMS. Only RCTs were included. Preclinical studies or Phase 1 studies, prognostic studies, retrospective studies, case reports, reviews, commentaries, and letters were excluded. Included trials had to have evaluated at least one of the injectable DMTs approved for use in clinical practice for the treatment of RRMS: IFN beta-1a, IFN beta-1b, GA, or peginterferon beta-1a. Doses evaluated were based on efficacy from clinical trials and in accordance with product insert labels.

Data extraction was carried out in parallel by two independent reviewers; discrepancies were reconciled by a third independent reviewer. Data elements extracted included study design, patient population characteristics, and efficacy and safety outcomes (ARR, EDSS scores, disability progression, discontinuations, and adverse events [AEs]). Specific efficacy outcomes of interest were ARR (measured at study endpoint), CDP3M and CDP6M (including onset of disability progression at the end of the randomized phase of the trials). For safety outcomes, the most common AEs (≥5% incidence in any treatment group) that were reported in the ADVANCE trial were extracted. In addition, AEs that occurred at an incidence of ≥3% in the treatment arms compared to the placebo group during Year 1 of the ADVANCE study were included (even if the overall incidence in the peginterferon beta-1a arm was <5%). As a conservative estimate in comparison with peginterferon beta-1a, the AEs included for comparators were only those reported in the ADVANCE trial. Safety outcomes from the ADVANCE trial were based on 2-year data and not adjusted for placebo results (placebo data was only collected for Year 1; in Year 2 placebo patients were re-randomized to receive peginterferon beta-1a). On this basis, the AEs of interest was the annual incidence of any AEs or serious AEs, and specifically arthralgia, back pain, depression, diarrhea, fatigue, flu-like symptoms, headache, injection-site reaction, leucopenia, nausea, pain in extremity, pruritus, and urinary tract infection. Although anti-IFN NAbs occurred in <1% of patients in either treatment arm over 2 years in the ADVANCE trial, antibody data were included in the safety analysis as these are common to IFN treatments [32].

Selection of trials for quantitative analyses was based on their connection to one or more trials to form a network for the meta-analysis. Studies with a duration of ≤6 months were excluded from quantitative analysis due to the inability of short-term studies to measure disease progression [33].

### Quality Assessment of Extracted Studies

The RCTs that met the inclusion criteria for the review were critically appraised for quality by means of a study grade and Jadad score [34]. The first measure assesses the adequacy of treatment allocation concealment, while the second measure examines study quality and study reporting [34]. Qualitative assessment of trials was also conducted using comprehensive assessment criteria based on the recommendations by the National Institute for Health and Care Excellence (NICE), the Cochrane critical appraisal tool, and the German Institute for Quality and Efficiency in Healthcare (IQWiG) guidelines [35–37]. Appraisal of studies was performed by two independent reviewers and discrepancies between reviewers were reconciled by a third independent reviewer. The majority of the studies were of good quality, although there is always an inherent bias associated with open label studies. Study quality was assessed and sensitivity analyses performed on the studies with inherent bias (see Table B in S1 Appendix for types of sensitivity analyses conducted).

### Statistical Analyses

NMAs were conducted using a set of Bayesian hierarchical models that used non-informative priors. The efficacy outcomes of interest for the analyses were ARR, CDP3M, and CDP6M. The model was used to estimate the relative effects of treatment with peginterferon beta-1a 125 μg every 2 weeks compared to IFN beta-1a 30 μg QW, IFN beta-1b 250 μg every other day (EOD), IFN beta-1a 22 μg three times a week (TIW), IFN beta-1a 44 μg TIW, GA 20mg OD, and placebo.

ARR was analyzed as a Poisson outcome using the total number of relapses observed within a treatment group out of the total person-time of follow-up for that treatment group calculated from study follow up. A Poisson regression was fit using log link function. Statistical methods and equations are described in Supplementary Appendix (Statistical Methods A in S1 Appendix).

Across studies there was heterogeneity in measured AE rates (varying AE definitions, AE measurements, and trial durations). Therefore, AE comparisons were calculated descriptively and based on annualized risks instead of applying statistics to the NMA methods, utilizing 2-year data and not adjusted for placebo results. The treatment-specific annual incidence of AEs or discontinuations was calculated using a weighted average across studies [38]. If an event occurred at a constant rate *r* per time unit *t*, then the probability that an event will occur during time *t* (provided the unit of time used in *r* and *t* are the same) was calculated as: *p* = 1-e^-rt^. The probability was then converted to a rate based on the equation: $r=-\frac{1}{t}\ln(1-p)$. Conducting NMAs for safety comparisons was not possible due to variant AE definitions, AE measurements, and trial durations across studies which would have introduced an unacceptable level of heterogeneity.

There is inevitable uncertainty around results inherent in a Bayesian NMA, which causes a certain amount of overlap in the credible intervals. Therefore, we evaluated rank probabilities and surface under the cumulative ranking curve (SUCRA) for all efficacy results of the NMAs to determine the probability that each treatment is the best among the given set of injectable treatments. Standard deviations in rank probabilities were a product of estimating probabilities from WinBUGS simulations. A higher probability of achieving rank 1 indicates a higher possibility that treated patients will experience a greater improvement in terms of the evaluated efficacy outcome.

### Model Fit and Heterogeneity

The treatment effect sizes were estimated using both fixed-effect and random-effect models. Model fit was evaluated using the overall residual deviance and deviance information criteria (DIC). The DIC is a general test for model fit (standard model fitting diagnostics) to assess and capture sensitivity to assumptions [39]; lower values of DIC suggests a more parsimonious model. A random-effects model was considered more appropriate due to the heterogeneity in patient and trial characteristics and hence constitutes the primary analysis (see Table C in S1 Appendix for fixed effect models efficacy outcomes, and Table D in S1 Appendix for a comparison between fixed- and random-effect models). For each outcome, one common heterogeneity parameter, tau^2^, was assumed across comparisons, which corresponded to the variance of underlying distribution. A tau^2^ value ≥1 is considered to indicate relatively high intra-study variability [40].

### Consistency

An assumption of NMA models is that direct and indirect sources of evidence estimate the same true treatment effect. This was evaluated by conducting conventional pairwise meta-analyses and testing consistency by comparing the direct and indirect evidence results to see if a statistically significant difference existed. We applied the back-calculation method to check for consistency within the evidence networks [41]. Based on the back-calculation method, the difference between direct and indirect estimate, ωAB=dABDir−dABRest was considered as an estimate of inconsistency. Our null hypothesis was that there was consistency between the direct and indirect evidence and we would reject the null hypothesis if there was a statistically significant difference between the direct and indirect evidence comparison (p<0.05). The test for consistency (ω = 0) between direct and indirect evidence suggested that there was evidence of inconsistency (p = 0.04) [42].

### Sensitivity Analyses

For each analysis, studies were fully assessed for baseline comparability. However, no study was excluded from the base-case analysis based on differences in the baseline characteristics of studies included in the analyses. Sensitivity analyses were conducted after omitting the studies with differences in study characteristics such as sample size (exclusion of studies with <50 patients per arm), blinding status (exclusion of studies with partial or assessor blinding or with unclear blinding status) and study duration (excluding studies <1 and >2 years for ARR analyses and estimate from Kaplan-Meir curve at 1 year for CDP endpoints) to check for robustness of the base-case analysis. Table B in S1 Appendix displays the sensitivity analysis conducted for the endpoints evaluated.

All NMAs were conducted using WinBUGS software, version 1.4.3.

## Results

### Systematic Review Results

**Fig 1:**
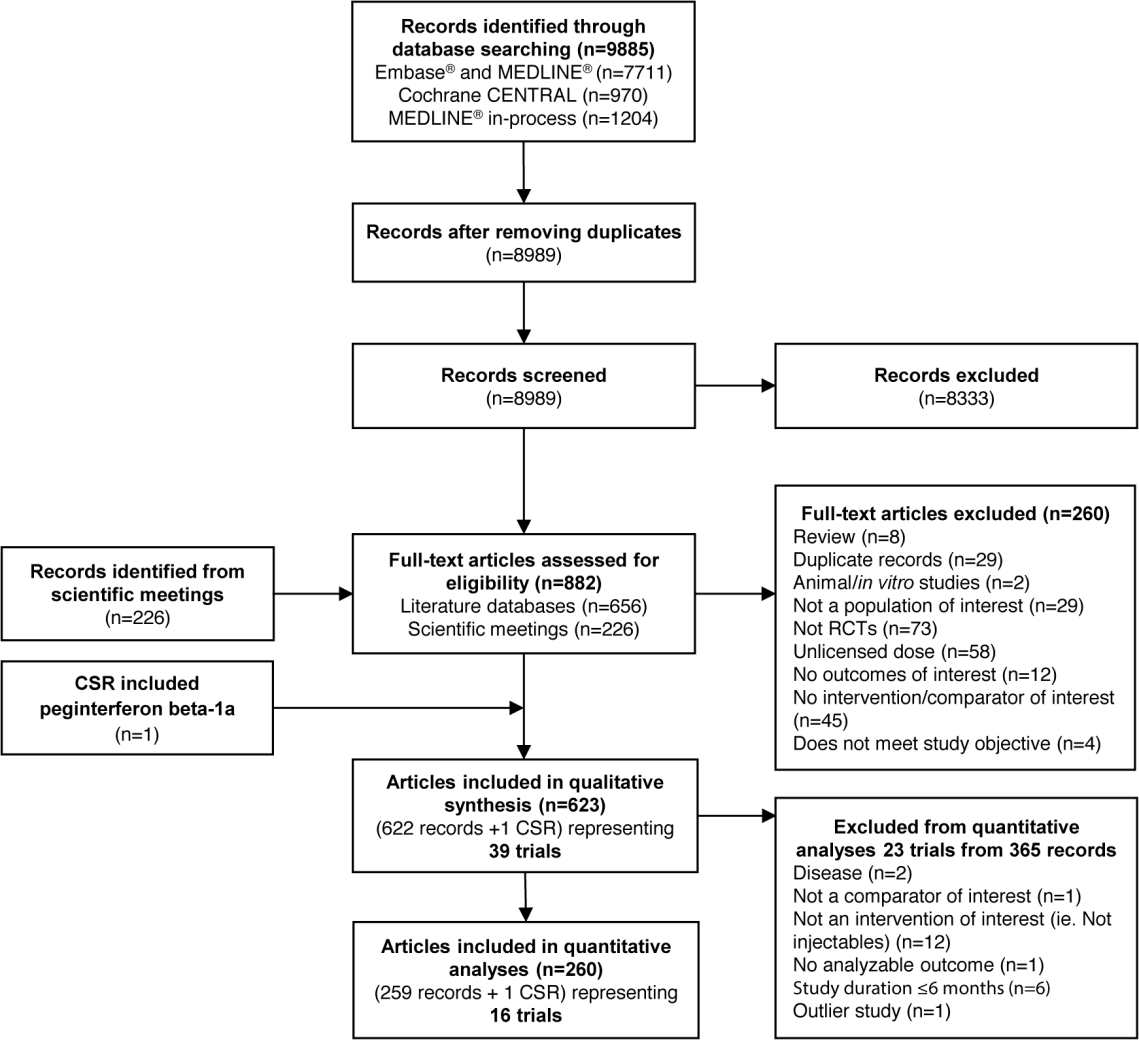
PRISMA Attrition Diagram for Systematic Literature Review. Abbreviations: CENTRAL, Cochrane Central Register of Controlled Trials; CSR, clinical study report, IFN, interferon; PRISMA, Preferred Reporting Items for Systematic Reviews and Meta-Analyses; PEG, pegylated; RCT, randomized controlled trial.

Results of the literature searches are presented in Fig 1 according to the Preferred Reporting Items for Systematic Reviews and Meta-Analyses (PRISMA) guidelines [43]. The 39 trials included in the qualitative portion of the systematic review were further evaluated for the feasibility of inclusion in quantitative analysis. Examination of patient characteristics across trials yielded no major differences. However, when examining study design, one trial (INCOMIN trial) was identified as an open-label trial that was not outcome assessor blinded, which can be associated with a high risk of bias. This study was therefore identified for further examination to assess the inconsistency of evidence [44]. Heterogeneity measured in between-study standard deviation was significantly reduced (from 0.28 to 0.22) when the INCOMIN trial was excluded from the NMA. Improved model fit was also demonstrated (DIC reduction from 108.05 to 96.18) when the INCOMIN trial was excluded from the NMA. For example, with the ARR endpoint, it was observed that the estimates from INCOMIN trial (IFN beta-1a 30 μg QW vs IFN beta-1a 44 mg TIW) were inconsistent when compared with indirect estimates (ω = 0.32, p = 0.04). Considering that the consistency assumption was not upheld with inclusion of the results of the INCOMIN trial, the trial was considered an outlier and hence excluded from the analysis. Table E in S1 Appendix displays the model fit statistics with and without INCOMIN data. A total of 16 RCTs (including a clinical study report, for which the data is now-published [Keiseier et al., 2014] [32]) were eventually included in the NMA (Table 1).

**Table 1:** Overview of Included Trials.

Trial	Treatment	Group (N)	Mean Age (SD) Years	% Female	% Caucasian	Disease Duration, Years Mean (SD)	EDSS Score at Baseline Mean (SD)	Number of Relapses 1 Year Prior to Baseline Mean (SD)
ADVANCE trial[24]	Peginterferon beta-1a 125 μg every 2 weeks	512	36.9 (9.8)	71	81.3	4.0 (5.09)	2.47 (1.26)	1.6 (0.67)
	Peginterferon beta-1a 125 μg every 4 weeks	500	36.4 (9.9)	70	82.4	3.4 (4.36)	2.48 (1.24)	1.5 (0.62)
	Placebo	500	36.3 (9.7)	72	-	3.5 (4.63)	2.44 (1.18)	1.6 (0.67)
BECOME trial[54]	GA 20 mg OD	39	36 (NR)	64	61.5	1.2 (0.2–34)*	2* (NR)	NR
	IFN beta-1b 250 μg EOD	36	36 (NR)	75	41.7	0.9 (0.1–24)*	2* (NR)	NR
BEYOND trial[53]	GA 20 mg OD	448	35.2 (NR)	68	90.6	5.1 (NR)	2.28 (NR)	1.6 (NR)
	IFN beta-1b 250 μg EOD	897	35.8 (NR)	70	92.5	5.3 (NR)	2.35 (NR)	1.6 (NR)
Bornstein 1987[56]	GA 20 mg OD	25	30 (NR)	56	92	4.9 (NR)	2.9 (NR)	NR
	Placebo	25	31 (NR)	60	100	4.6 (NR)	3.2 (NR)	NR
BRAVO trial[68]	IFN beta-1a 30 μg QW	447	38.5* (NR)	68.7	-	5.3* (NR)	2.5* (NR)	1.0* (NR)
	Placebo	450	37.5* (NR)	71.3	-	4.7* (NR)	2.5* (NR)	1.0* (NR)
Calabrese 2011[46]	GA 20 mg OD	48	38.9 (10.2)	73	-	5.5 (6.1)	2.1 (1.1)	NR
	IFN beta-1a 30 μg QW	47	34.8 (9.6)	68	-	5.3 (5.1)	1.9 (0.8)	NR
	IFN beta-1a 44 μg TIW	46	35.9 (9.1)	70	-	5.7 (4.9)	1.9 (1)	NR
CombiRx trial[51]	GA 20 mg OD	259	39 (9.5)	69	90.3	1 (2.9)	1.9 (1.2)	1.6 (0.7)
	IFN beta-1a 30 μg QW	250	37.6 (10.2)	71	84.8	1.4 (4)	2 (1.2)	1.7 (0.9)
CONFIRM trial*[52]	GA 20 mg OD	350	36.7 (9.1)	71	82.9	4.4 (4.7)	2.57 (1.22)	1.4 (0.64)
	Placebo	363	36.9 (9.2)	69	84.0	4.8 (5.01)	2.59 (1.17)	1.4 (0.8)
Copolymer 1 MS trial[57]	GA 20 mg OD	125	34.6 (6.0)	70	94.4	7.25 (4.85)	2.82 (1.19)	NR
	Placebo	126	34.3 (6.5)	76	93.7	6.64 (5.09)	2.42 (1.28)	NR
Etemadifar 2006[50]	IFN beta-1a 30 μg QW	30	28.1 (1.2)	80	-	2.9 (2.3)	1.9 (1.1)	2 (0.8)
	IFN beta-1a 44 μg TIW	30	27.4 (1.2)	77	-	3 (2.2)	2.1 (1)	2.4 (1)
	IFN beta-1b 250 μg EOD	30	29.9 (1.4)	70	-	3.7 (2.3)	1.9 (0.7)	2.2 (0.7)
European and Canadian glatiramer trial[58]	GA 20 mg OD	113	34.1 (7.4)	77	-	7.9 (5.5)	2.3 (1.1)	NR
	Placebo	114	34 (7.5)	73	-	8.3 (5.5)	2.4 (1.2)	NR
EVIDENCE trial[49]	IFN beta-1a 30 μg QW	338	37.4	75	89.6	6.7 (NR)	2.3 (NR)	NR
	IFN beta-1a 44 μg TIW	339	38.3	75	92.3	6.5 (NR)	2.3 (NR)	NR
IFNB MS trial[47]	IFN beta-1b 250 μg EOD	124	35.2	69	93.6	4.7 (NR)	3 (NR)	NR
	Placebo	123	36	72	94.3	3.9 (NR)	2.8 **(**NR)	NR
MSCRG Trial[55]	IFN beta-1a 30 μg QW	158	36.7	75	93.0	6.6 (NR)	2.4 (0.8)	NR
	Placebo	143	36.9	72	91.6	6.4 (NR)	2.3 (0.8)	NR
PRISMS trial[48]	IFN beta-1a 22 μg TIW	189	34.8*	67	-	5.4*	2.5 (1.2)	NR
	IFN beta-1a 44 μg TIW	184	35.6*	66	-	6.4*	2.5 (1.3)	NR
	Placebo	187	34.6*	75	-	4.3*	2.4 (1.2)	NR
REGARD trial[59]	GA 20 mg OD	378	36.8 (9.5)	72	93.9	-	2.33 (1.31)	NR
	IFN beta-1a 44 μg TIW	386	36.7 (9.8)	69	93.3	3.7	2.35 (1.28)	1*

*Median and/or range.

Abbreviations: EDSS, Expanded Disability Status Scale; EOD, every other day; GA, glatiramer acetate; IFN, interferon; μg, microgram; N, evaluable patients; N, number of patients with event; NR, not reported; OD, once daily; PEG, pegylated; QW, once a week; RCT, randomized controlled trial; SD, standard deviation; TIW, 3 times a week

In terms of quality assessment, all 16 trials were randomized, but only 11 trials reported the randomization method and treatment allocation concealment. The majority of trials (15 of 16) were blinded appropriately to avoid detection bias, and there were no major imbalances in the baseline characteristics of the treatment groups. All but one trial analyzed outcomes on an intention-to-treat basis [45]. The results of the sensitivity analysis on blinding status were not affected by the quality of trials. Due to the small number of studies evaluating similar interventions and comparisons, it was not possible to assess publication bias using funnel plots. Details of the quality assessment are presented in the Supplementary Appendix (Table F, Figs A-B in S1 Appendix).

The characteristics of the trials included in the analysis are presented in Table 1. Baseline patient characteristics were similar across trials and treatments. The mean age across trials ranged from 29–39 years, and the majority of participants were female and Caucasian. There were variations in the mean disease duration across trials, with values ranging from 1–8.3 years. Similarly, there were variations in the definition of relapse across trials, particularly the duration of symptoms. In some trials, the time cut-off was “at least 24 hours,” while in others it was “at least 48 hours.”

Of the 16 trials included in the analysis, nine defined *relapse* as the appearance of a new neurological symptom or worsening of an old symptom lasting at least 24 hours [24,46–53]. Five trials required a duration lasting at least 48 hours [50–54], and two trials did not specify the duration [45,59]. All of the trials defining *relapse* as lasting for at least 48 hours referred to the appearance of more than one new, or worsening of more than one old, neurological symptom, whereas the majority of the trials using the other definition refer to the occurrence of one new, or worsening of one old, neurological symptom.

### Network Meta-Analysis

**Fig 2:**
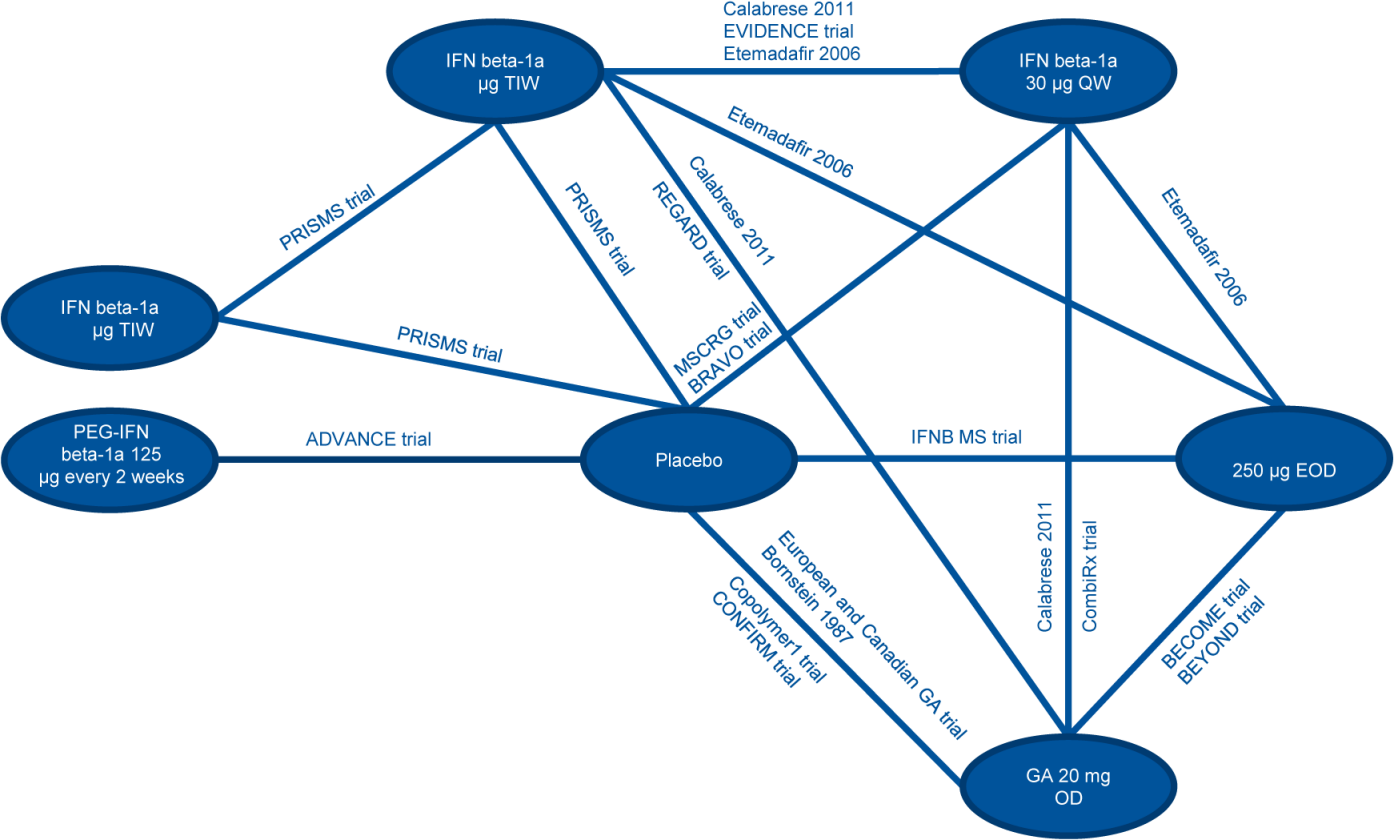
Network Diagram for ARR. Abbreviations: ARR, annualized relapse rate; EOD, every other day; GA, glatiramer acetate; IFN, interferon; OD, once daily; PEG, pegylated; QW, once a week; TIW, 3 times a week.

**Fig 3:**
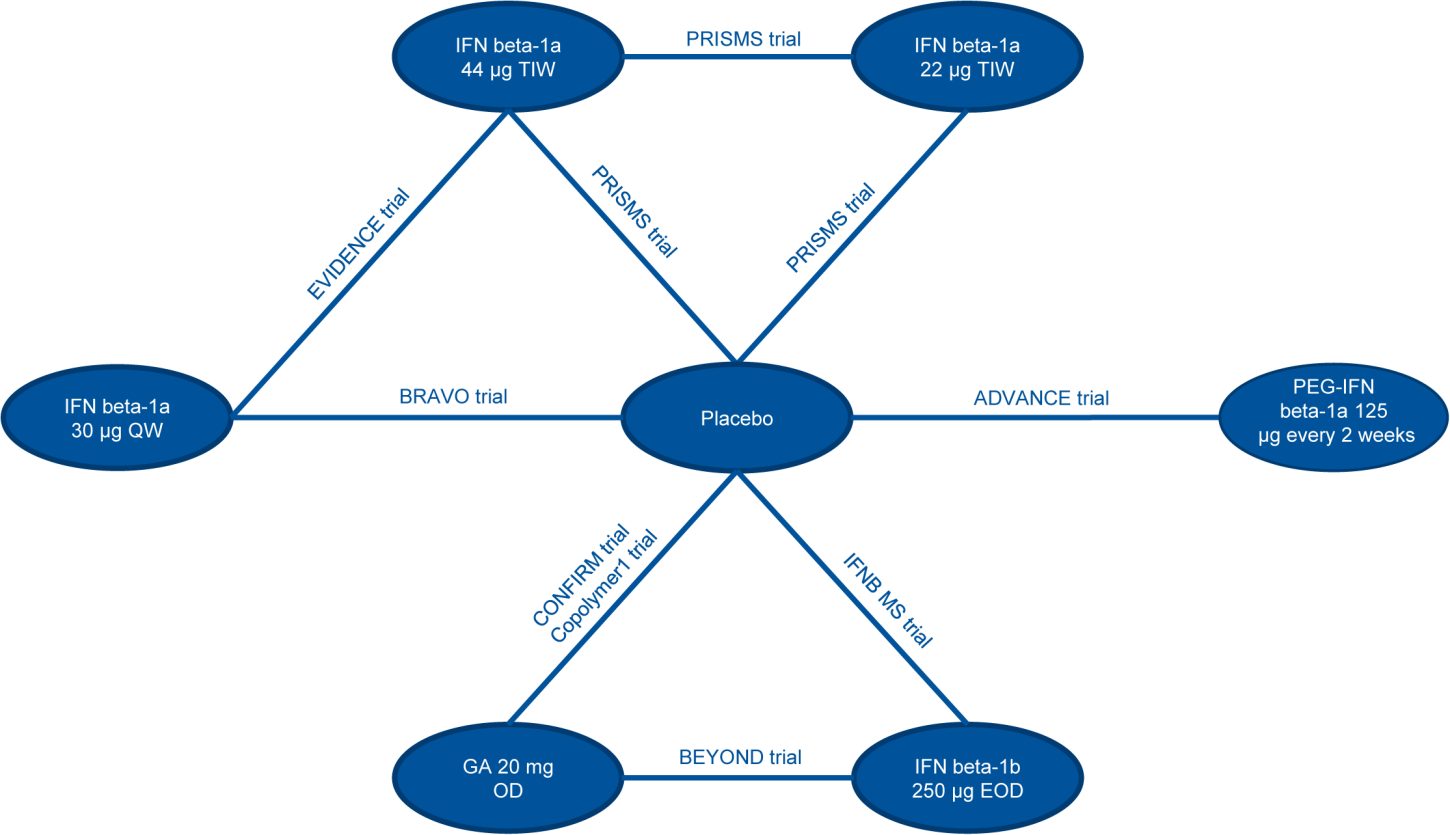
Network Diagram for CDP3M. Abbreviations: CDP3M, 3-month confirmed disability progression; EOD, every other day; GA, glatiramer acetate; IFN, interferon; OD, once daily; PEG, pegylated; QW, once a week; TIW, 3 times a week.

**Fig 4:**
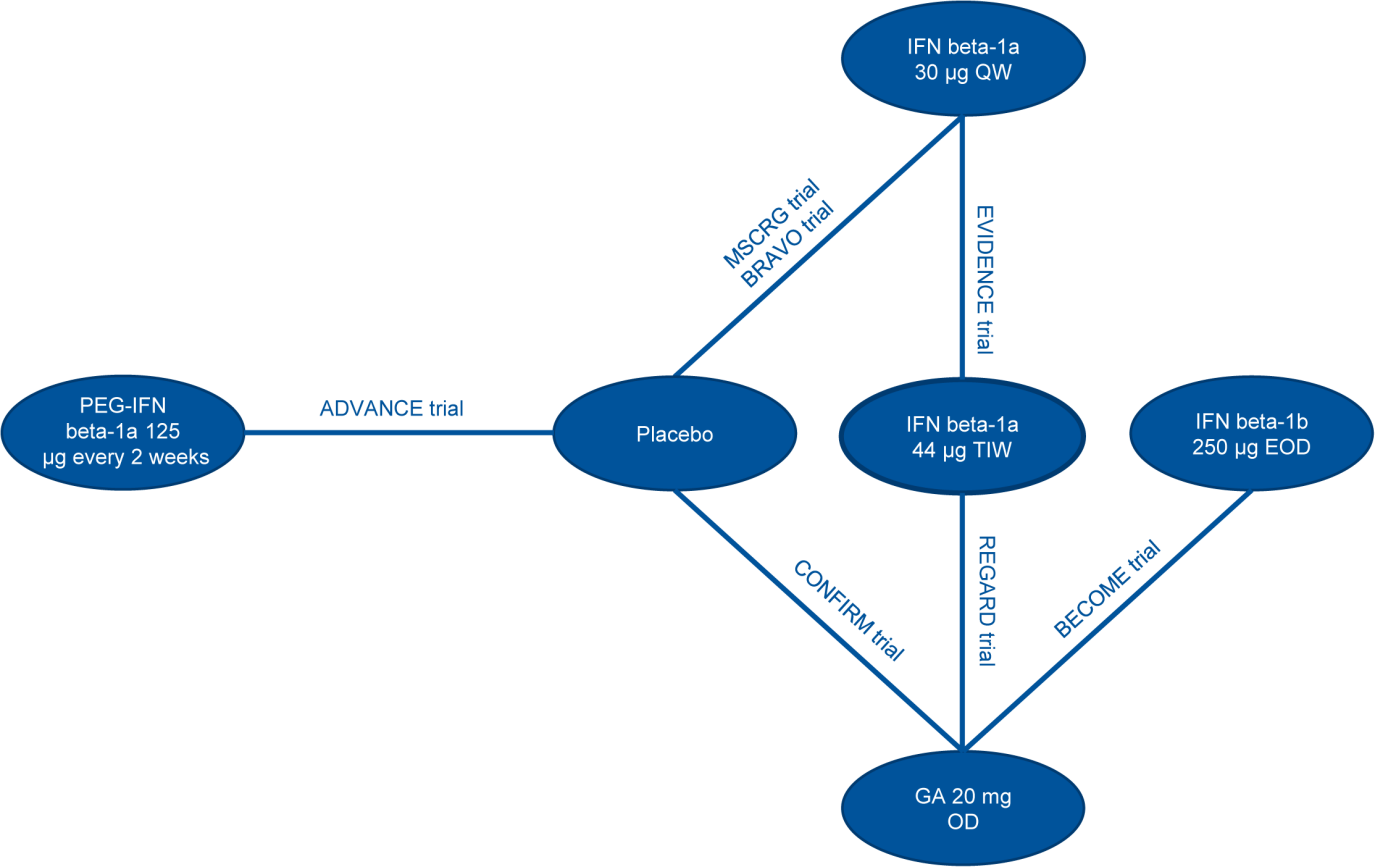
Network Diagram for CDP6M. Abbreviations: CDP6M, 6-month confirmed disability progression; EOD, every other day; GA, glatiramer acetate; IFN, interferon; OD, once daily; PEG, pegylated; QW, once a week; TIW, three times a week.

Input data used for the analyses are presented in the Supplementary Appendix (Tables G-I in S1 Appendix). The network diagrams of treatments included in the analysis are presented in Figs 2, 3 and 4, for ARR, CDP3M, and CDP6M, respectively. While the primary objective of the NMA was to provide a comparison between peginterferon beta-1a and well-established DMTs, NMA results comparing all treatment combinations are provided in the Supplementary Appendix (Tables J-L in S1 Appendix).

#### Annualized Relapse Rates

**Fig 5:**
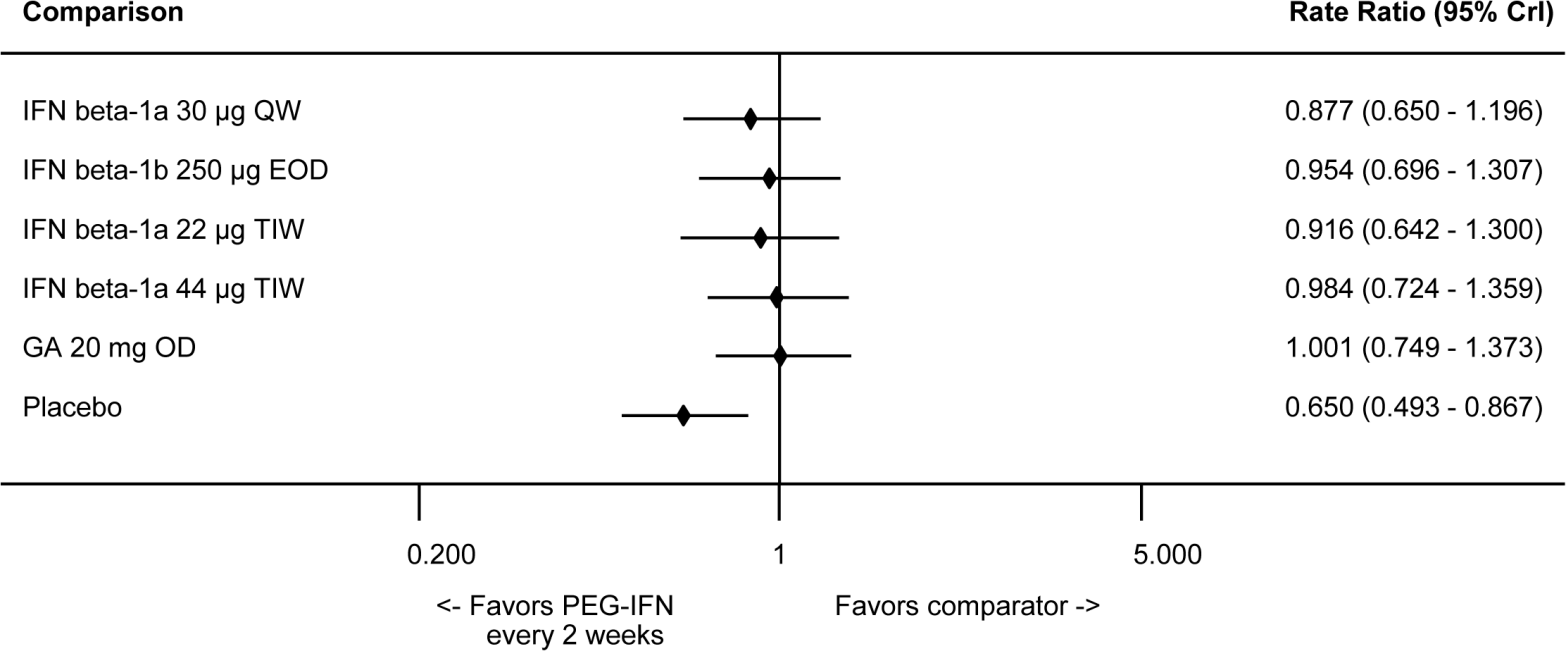
Summary Plot Showing Relative ARR of Peginterferon Beta-1a vs Other Injectables (RR and 95% CrI). Effect size <1 indicates favorable efficacy of intervention. Abbreviations: ARR, annualized relapse rate; CrI, credible interval; EOD, every other day; GA, glatiramer acetate; IFN, interferon; OD, once daily; PEG, pegylated; QW, once a week; RR, rate ratio; TIW, 3 times a week.

In the NMA, peginterferon beta-1a 125 μg every 2 weeks was associated with a statistically significant improvement in ARR when compared to placebo and a numerical improvement compared to all IFNs, although none of the comparisons with active comparators were statistically significant (Fig 5). RRs and 95% credible intervals derived from the NMA for peginterferon beta-1a 125 μg every 2 weeks were 0.88 (0.65–1.20) vs. IFN beta-1a 30 μg QW, 0.95 (0.70–1.31) vs. IFN beta-1b 250 μg EOD, 0.92 (0.64–1.30) vs. IFN beta-1a 22 μg TIW, 0.98 (0.72–1.36) vs. IFN beta-1a 44 μg TIW, 1.00 (0.75–1.37) vs. GA 20 mg OD, and 0.65 (0.49–0.87) vs. placebo. A comparison for the placebo group across all studies is provided in the Supplementary Appendix (Fig C in S1 Appendix).

The assessment of rank probabilities indicated that peginterferon beta-1a presented the greatest likelihood of improving ARR, at 42.25%, among all the evaluated injectable treatments (Table 2). Results for the standard deviations of the rank probabilities as a measure of uncertainty are provided in the Supplementary Appendix (Table M in S1 Appendix).

**Table 2:** Rank Probability of Best Outcomes by Treatment for ARR.

	Rank 1	Rank 2	Rank 3	Rank 4	Rank 5	Rank 6	Rank 7	SUCRA
Placebo	0.00	0.00	0.00	0.00	0.01	0.56	99.43	0.00
IFN beta-1a 30 μg QW	0.34	1.60	4.56	13.91	30.76	48.81	0.02	0.30
IFN beta-1b 250 μg EOD	6.58	15.21	23.32	26.67	20.16	8.05	0.01	0.56
IFN b-1a 22 μg TIW	6.75	9.05	12.76	18.69	27.95	24.57	0.23	0.46
IFN beta-1a 44 μg TIW	17.99	27.00	25.65	20.84	7.58	0.94	0.00	0.71
GA 20 mg OD	26.09	36.28	24.65	9.90	2.44	0.64	0.00	0.79
PEG IFN beta-1a 125 μg every 2 weeks	42.25	10.86	9.06	10.00	11.09	16.43	0.31	0.69

Abbreviations: ARR, annualized relapse rate; CDP3M, 3-month confirmed disability progression; CDP6M, 6-month confirmed disability progression; EOD, every other day; GA, glatiramer acetate; IFN, interferon; μg, microgram; OD, once daily; PEG, pegylated; QW, once a week; SUCRA, surface under the cumulative ranking curve; TIW, 3 times a week

#### 3-Month Confirmed Disability Progression

**Fig 6:**
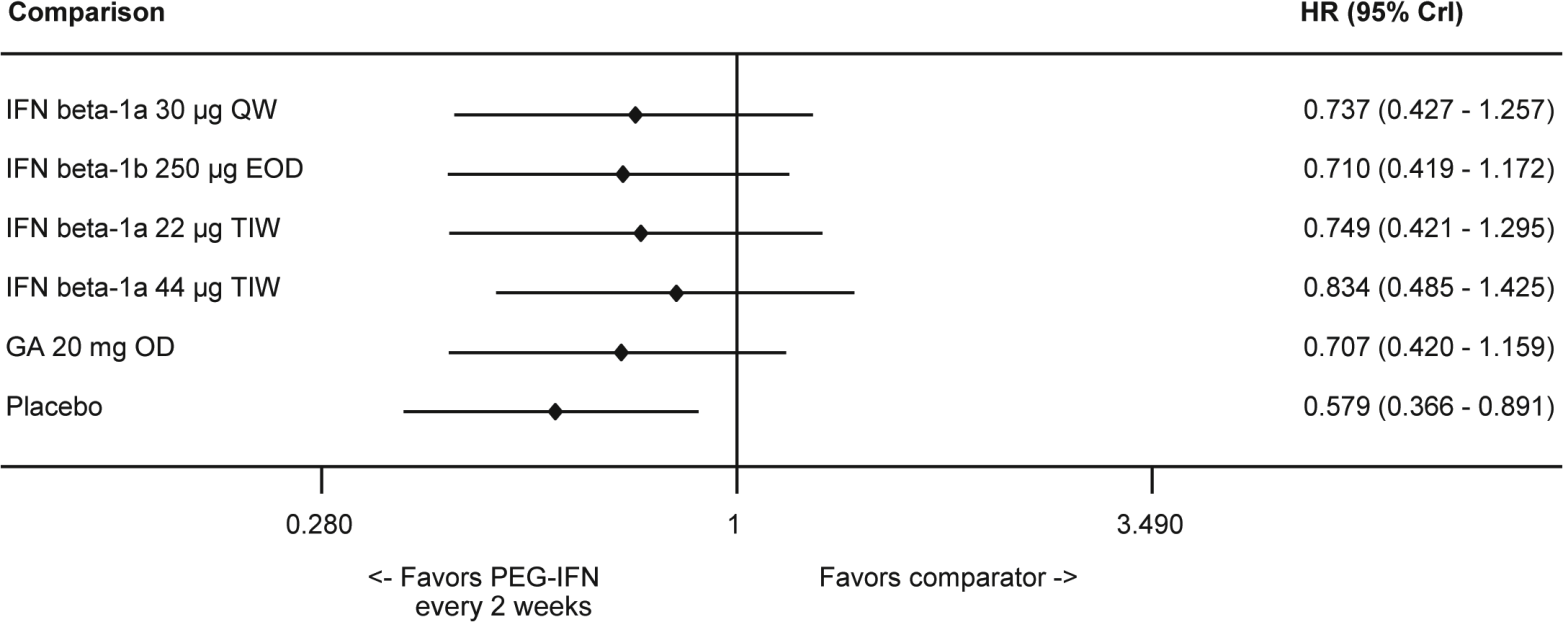
Summary Plot Showing the CDP3M for Peginterferon Beta-1a vs Comparators (HR and 95% CrI). Effect size <1 indicates favorable efficacy of intervention. Abbreviations: CDP3M, 3-month confirmed disability progression; CrI, credible interval; EOD, every other day; GA, glatiramer acetate; HR, hazard ratio; IFN, interferon; OD, once daily; PEG, pegylated; QW, once a week; TIW, 3 times a week.

Peginterferon beta-1a was numerically superior to evaluated injectable DMTs in terms of CDP3M, but statistical significance was not reached (Fig 6). HRs and 95% credible intervals derived from the NMA for peginterferon beta-1a 125 μg every 2 weeks were 0.74 (0.43–1.26) vs. IFN beta-1a 30 μg QW, 0.71 (0.42–1.17) vs. IFN beta-1b 250 μg EOD, 0.75 (0.42–1.30) vs. IFN beta-1a 22 μg TIW, 0.83 (0.49–1.43) vs. IFN beta-1a 44 μg TIW, 0.71 (0.42–1.16) vs. GA 20 mg OD, and 0.58 (0.37–0.89) vs. placebo. Similar to the NMA, peginterferon beta-1a was associated with a statistically significant difference in CDP3M compared to placebo in the ADVANCE trial [24]. A comparison for the placebo group across all studies is provided in the Supplementary Appendix (Table N in S1 Appendix).

The likelihood of achieving rank 1 for the improvement of CDP3M was highest with peginterferon beta-1a, 67.17%, among all the injectable treatments (Table 3). Results for the standard deviations of the rank probabilities as a measure of uncertainty are provided in the Supplementary Appendix (Fig D in S1 Appendix).

**Table 3:** Rank Probability of Best Outcomes by Treatment for CDP3M.

	Rank 1	Rank 2	Rank 3	Rank 4	Rank 5	Rank 6	Rank 7	SUCRA
Placebo	0.00	0.01	0.09	0.49	3.97	17.79	77.64	0.05
IFN beta-1a 30 μg QW	4.14	13.77	20.90	21.72	16.64	17.57	5.27	0.49
IFN beta-1b 250 μg EOD	2.32	9.93	14.24	20.77	26.52	20.62	5.61	0.43
IFN beta-1a 22 μg TIW	6.75	17.64	19.93	19.14	13.97	16.73	5.85	0.52
IFN beta-1a 44 μg TIW	17.94	36.73	21.62	12.14	7.92	3.25	0.41	0.72
GA 20 mg OD	1.68	8.39	14.83	20.71	28.03	21.60	4.76	0.42
PEG IFN beta-1a 125 μg every 2 weeks	67.17	13.54	8.39	5.04	2.95	2.44	0.46	0.88

Abbreviations: ARR, annualized relapse rate; CDP3M, 3-month confirmed disability progression; CDP6M, 6-month confirmed disability progression; EOD, every other day; GA, glatiramer acetate; IFN, interferon; μg, microgram; OD, once daily; PEG, pegylated; QW, once a week; SUCRA, surface under the cumulative ranking curve; TIW, 3 times a week

#### 6-Month Confirmed Disability Progression

**Fig 7:**
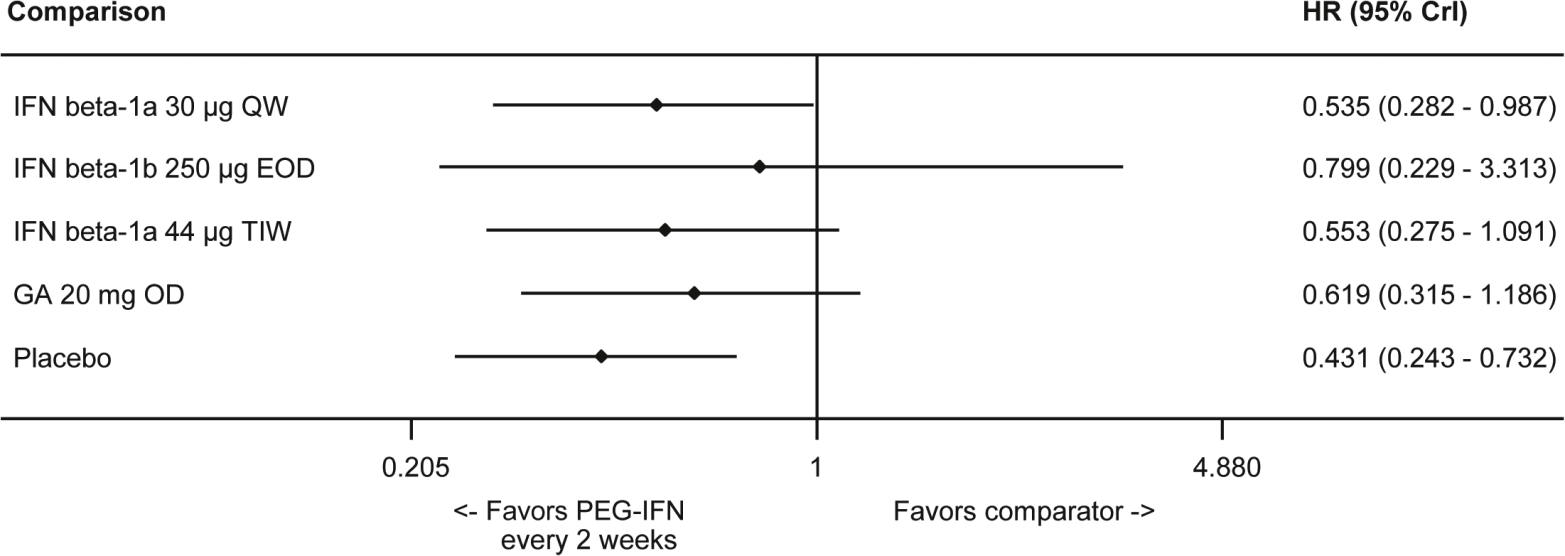
Summary Plot Showing the CDP6M for Peginterferon Beta-1a vs Comparators (HR and 95% CrI). Effect size <1 indicates favorable efficacy of intervention. Abbreviations: CDP6M, 6-month confirmed disability progression; CrI, credible interval; EOD, every other day; GA, glatiramer acetate; HR, hazard ratio; IFN, interferon; OD, once daily; PEG, pegylated; QW, once a week; TIW, 3 times a week.

As described in the Methods section, inconsistency in evidence was judged among direct and indirect difference. It was observed that the estimates from INCOMIN trial (IFN beta-1a 30 μg QW vs IFN beta-1b 250 μg EOD contributed to inconsistent estimates for (IFN beta-1a 30 μg QW vs placebo; ω = 0.19, p<0.001). Considering that the consistency assumption was not true with the results of INCOMIN trial, this trial was considered an outlier and it was excluded from the analysis. Peginterferon beta-1a 125 μg every 2 weeks was associated with a statistically significant reduction in the likelihood of CDP6M when compared to placebo and IFN beta-1a 30 μg QW, but the reduction was non-significant compared to other evaluated DMTs based on the NMA results (Fig 7). HRs and 95% credible intervals derived from the NMA for peginterferon beta-1a 125 μg every 2 weeks were 0.54 (0.28–0.99) vs. IFN beta-1a 30 μg QW, 0.80 (0.23–3.31) vs. IFN beta-1b 250 μg EOD, 0.55 (0.28–1.09) vs. IFN beta-1a 44 μg TIW, 0.62 (0.32–1.19) vs. GA 20 mg OD, and 0.43 (0.24–0.73) vs. placebo. A comparison for the placebo group across all studies is provided in the Supplementary Appendix (Fig E in S1 Appendix).

The likelihood of achieving rank 1 for improving CDP6M was highest for peginterferon beta-1a, 57.80%, among the evaluated injectable treatments (Table 4). Results for the standard deviations of the rank probabilities as a measure of uncertainty are provided in the Supplementary Appendix (Table O in S1 Appendix).

**Table 4:** Rank Probability of Best Outcomes by Treatment for CDP6M.

	Rank 1	Rank 2	Rank 3	Rank 4	Rank 5	Rank 6	SUCRA
Placebo	0.00	0.10	0.87	4.43	21.07	73.54	0.1
IFN beta-1a 30 μg QW	0.50	8.13	20.23	30.53	36.27	4.35	0.4
IFN beta-1b 250 μg EOD	37.76	25.56	8.08	6.89	8.46	13.26	0.7
IFN beta-1a 44 μg TIW	1.50	10.27	23.15	33.28	23.85	7.96	0.4
GA 20 mg OD	2.45	22.15	42.74	22.55	9.24	0.87	0.6
PEG IFN beta-1a 125 μg every 2 weeks	57.80	33.79	4.93	2.33	1.11	0.04	0.9

Abbreviations: ARR, annualized relapse rate; CDP3M, 3-month confirmed disability progression; CDP6M, 6-month confirmed disability progression; EOD, every other day; GA, glatiramer acetate; IFN, interferon; μg, microgram; OD, once daily; PEG, pegylated; QW, once a week; SUCRA, surface under the cumulative ranking curve; TIW, 3 times a week

#### Sensitivity Analyses

The results of the main analysis did not differ significantly from that of the sensitivity analyses based on study duration, blinding status of trials, and sample size for the evaluated outcomes. There was no change in the direction or material difference in the magnitude of the relative estimates of comparison between peginterferon and other interventions with regard to ARR, CDP3M, and CDP6M (results presented Tables P-R, respectively, in S1 Appendix). Additionally, the quality of trials based on the risk of bias assessment described earlier did not impact the results of the analyses.

### Adverse Events

**Fig 8:**
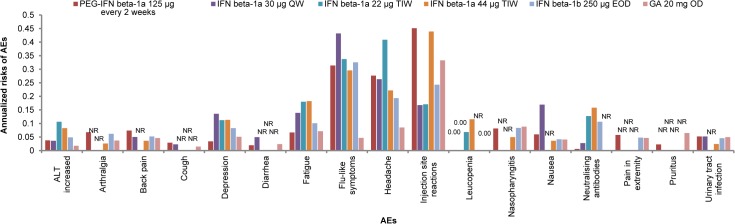
Annual Risk of AEs by Treatment. Abbreviations: AE, adverse event; ALT, aminotransferase; GA, glatiramer acetate; IFN, interferon; NR = not reported; PEG, pegylated.

Comparison of AEs was not possible within the NMA. However, based on a non-meta-analyzed comparison the safety and tolerability profile of peginterferon beta-1a 125 μg every 2 weeks appears consistent with that of other evaluated treatments, with no evidence for additional AE burden. For AEs associated with the IFN class the annualized risk is within the expected range. In general, risk of AEs is lower for peginterferon beta-1a compared to the other injectable platform treatments for the AEs evaluated (Fig 8).

Annualized risk of injection-site reactions, the most frequently reported AE for peginterferon beta-1a, is similar between peginterferon beta-1a and IFN beta-1a 44 μg TIW, and higher than those reported for other IFNs and GA. However, similar to IFNs and GA, the majority of patient-reported injection site reactions with peginterferon beta-1a were mild or moderate, with only 3% of patients reporting severe injection-site reactions over 2 years of treatment [60].

## Discussion

The objectives of the analysis were to evaluate the relative efficacy and annualized safety risks of subcutaneous peginterferon beta-1a and other approved injectable DMTs for the treatment of RRMS. The NMA methodology was used to derive relative estimates by combining direct and indirect evidence in the absence of robust evidence from head-to-head comparisons in clinical trials. Previous MS therapy studies have utilized MTCs and NMAs to evaluate the efficacy of fingolimod and natalizumab versus first-line therapies [61,62], to evaluate safety and efficacy of IFNs, GA, teriflunomide, natalizumab, fingolimod, and mitoxantrone [63], and to evaluate efficacy and rank MS treatments including immunosuppressants, immunomodulators, and monoclonal antibodies [64]. To our knowledge, however, this is the first indirect comparison of peginterferon beta-1a and other injectable DMTs.

Results of the analyses showed that peginterferon beta-1a has at least comparable efficacy to other approved injectable DMTs (e.g., IFN beta treatments and GA) and superior efficacy compared to placebo as defined by relapse reduction and reduction in CDP3M. Peginterferon beta-1a had a statistically superior efficacy profile compared to IFN beta-1a 30 μg QW and placebo for CDP6M. The comparison with other active comparators yielded numerically higher but mostly non-significant differences in efficacy. It should be noted that the credible intervals were narrow for ARR, suggesting a strong comparable efficacy, while the intervals were wider for disease progression outcomes, suggesting that there was more uncertainty regarding comparable outcomes and that head-to-head trials may detect a benefit for peginterferon beta-1a. The majority of trials are two years in duration; while available efficacy data for peginterferon beta-1a in this NMA are from placebo-controlled Year 1 of the ADVANCE study. Although an additional reduction in ARR for peginterferon beta-1a has been observed in Year 2 of the ADVANCE study [25], it is unknown whether ARR also has the potential to be further reduced in the placebo arm if continued in to Year 2 (all patients were re-randomized to active treatment at the end of Year 1). This caveat should be considered when annualizing the data from Year 1 of ADVANCE and comparing to trials of longer durations. However, even with a 1-year data, peginterferon beta-1a showed a statistically significant difference versus placebo for CDP3M and CDP6M, suggesting a significant advantage in efficacy of peginterferon beta-1a compared with placebo [24,25]. Additionally, it is unclear if the 2-year duration of the majority of the trials is sufficient to detect statistically significant differences in disability progression among active treatments, which may explain why statistically significant differences between peginterferon beta-1a and injectable comparators were not seen. Lastly, the burden of injection frequency and how it relates to compliance and the potential impact on long-term efficacy was not analyzed in the current study, as it is not reported in the included RCTs. Real-world evidence suggests that as administration frequency decreases, patient compliance increases due to reduced administration fatigue [65]. It can be hypothesized that the injectable DMT with the lowest dose frequency, peginterferon beta-1a, would be associated with fewer injection-related AEs, would improve patient adherence to treatment, and would be a valuable treatment option. The link between dosing frequency and adherence to treatment is well established [66,67], and is an important consideration in instituting care management plans for patients with MS. Patients requiring less frequent dosing, are more likely to be adherent to treatment and therefore clinical trial results align more closely with real-world efficacy [21], and derive substantial improvement in their quality of life [23]. Of note is the full compliance of >99% of patients in the ADVANCE study [24].

Given the heterogeneity across studies in the measurement of AEs, which was assessed descriptively rather than statistically, a formal NMA comparison is not meaningful. Although safety outcomes were not evaluated within the NMA, AEs were descriptively assessed using annualized risk. Peginterferon beta-1a had a safety profile that was comparable to other injectable treatments, as measured by annualized risk of AEs, and specifically to other IFN treatments as measured by annualized risk and type of AEs (flu-like symptoms incidence similar to other IFNs, and injection-site reactions similar to other subcutaneous IFNs and GA). Additionally, peginterferon beta-1a had a lower incidence of NAbs than any other DMT evaluated. Presented data does not capture frequency of AEs, which is important to note for AEs that are related to injection frequency such as flu-like symptoms and injection-site reactions. Peginterferon beta-1a reduces the annual injection frequency by up to 93%, which suggests that patients would experience fewer injection related events. No new safety concerns were found for peginterferon beta-1a compared to other injectable DMTs. Considering the types and rates of AEs associated with other approved injectable DMTs, peginterferon beta-1a is a suitable alternative to be considered in the treatment of RRMS. Although no statistical comparisons across studies are made, results should be interpreted with caution given the heterogeneity and potential biases.

### Limitations

Our study has several limitations. First, there were variations in the definition of relapse across trials, and the analyses assumed that relative treatment effects were similar across studies, regardless of the definition of relapse. Although this increased the robustness of the evidence network, it could limit the inferences drawn from the analysis. However, the effect of varying definitions is delimited by the consistency of the results observed in the analyses.

Secondly, in conducting this NMA, as with other indirect treatment comparison analyses, we have assumed that the populations are sufficiently similar to ensure comparability. Additionally, we have assumed that, whatever differences exist, occur randomly due to natural variation rather than potentially unmeasured, inherent differences between the trials and their populations. In this analysis, although baseline characteristics appeared similar, a sensitivity analysis was performed which found there was a low risk of bias across studies 15 of 16 studies (results from one study were not clear). Regardless, a degree of heterogeneity in trial results should be expected and accounted for in the precision of estimates. Moreover, NMAs can be considered observational studies and, as such, residual confounding will always be present. Systematic differences in characteristics among trials in a network can bias indirect comparison results. Results of the sensitivity analysis involving exclusion of trials based on certain characteristics were not different from that of the main analysis, which included all trials. Although not all possible sensitivity analyses were performed, key sensitivity analyses were carried out, specifically analyses regarding the exclusion of heterogeneous studies.

Thirdly, clinical trial programs in MS are not powered to detect statistically significant differences in safety outcomes between treatment arms due to varying AE definitions, AE measurements, and trial durations across studies, therefore an NMA was not conducted. Instead, annualized risk was calculated. Included AEs were identified through data on file from the ADVANCE study, based on pre-defined criteria. This is important because an AE not reported is not equivalent to zero risk of events. Although the results provide some insight into the safety profiles of evaluated treatments, they should be interpreted with caution. Differences between data sources, variations in patient population mixes (naïve vs experienced), definitions of AEs, and high rates of AEs in placebo arms may have also affected the safety analyses. Further, frequencies of injection-site reaction and flu-like symptoms may be correlated with injection frequency, suggesting that the reduced injection frequency offered by peginterferon beta-1a 125 μg every 2 weeks may alleviate the prevalence of these AEs. Additionally, some AEs are time-dependent (e.g. flu-like symptoms) and their incidence decreases over the course of treatment. Annual rates from the complete 2-year ADVANCE study data may be needed to accurately interpret the indirect trial comparisons. An extension trial, ATTAIN, is currently underway with the aim of evaluating the long-term safety and efficacy of peginterferon beta-1a in patients with RRMS.

Fourthly, due to having full access to AE data for peginterferon beta-1a, AEs were identified in a conservative manner, which was not necessarily the case with other clinical studies for comparator treatments. Hence, for peginterferon beta-1a patients with any of the multiple types of injection-site reactions (by MedDRA system organ class and preferred term), the most common AEs for peginterferon beta-1a were included but it is likely that comparator drug studies were less inclusive of injection site reaction criteria. Additional Phase 4 clinical trials for peginterferon beta-1a are currently underway to further assess the impact of injection site reactions on patients administered peginterferon beta-1a.

Finally, head-to-head trials provide the highest level of evidence when comparing interventions. However, the comparison in most head-to-head trials is often against placebo as opposed to another active treatment. As such, evidence from an indirect treatment comparison provides the next best evidence for the relative differences between treatments in the absence of direct comparisons between active treatments.

## Conclusion

Based on the evidence from the systematic literature review and NMA, peginterferon beta-1a demonstrated comparable efficacy compared to non-pegylated IFNs and GA in the treatment of RRMS. In addition, based on the descriptive analysis of relative safety data, peginterferon beta-1a is well-tolerated and has the potential to reduce the frequency of some of the more prevalent AEs associated with most injectable DMTs, such as flu-like symptoms and injection-site reactions. The efficacy profile, the lower injection frequency, and a consistently more favorable safety profile of the peginterferon beta-1a 125 μg every 2 weeks regimen make it a suitable alternative to other approved injectable DMTs for the treatment of patients with RRMS.

## Supporting Information

S1 Appendix Supporting Information appendix. SI_Caption>
(DOCX)

S1 Dataset WinBUGS code used to conduct the analysis (fixed effect). (ZIP)

S2 Dataset WinBUGS code used to conduct the analysis (random effects). (ZIP)

S3 Dataset Raw data used to calculate annualized risk of AEs. (XLSX)

S4 Dataset Model fit summary statistics of CDL6M using vague and weakly informative priors (XLSX)

S1 PRISMA Checklist Completed Prisma checklist. (DOC)
