# Supplementary material for: A Network Meta-Analysis of Efficacy and Evaluation of Safety of Subcutaneous Pegylated Interferon Beta-1a versus Other Injectable Therapies for the Treatment of Relapsing-Remitting Multiple Sclerosis
Source: PLoS One. 2015 Jun 3;10(6):e0127960. doi: 10.1371/journal.pone.0127960 (PMC4454514; doi:10.1371/journal.pone.0127960)
Supplement: S1 Appendix — SI_Caption> (DOCX) [file pone.0127960.s001.docx]

**Supplementary Appendix**

Table A. Embase and MEDLINE Search Strategy for Randomized Controlled Trials (Searches Conducted on March 27, 2014)

|  | Search Terms | Results |
| --- | --- | --- |
| #1 | 'clinical trial'/exp | 1 130 835 |
| #2 | 'randomization'/de | 75 215 |
| #3 | 'controlled study'/de | 4 418 477 |
| #4 | 'comparative study'/de | 1 021 373 |
| #5 | 'single blind procedure'/de | 17 756 |
| #6 | 'double blind procedure'/de | 113 975 |
| #7 | 'crossover procedure'/de | 38 194 |
| #8 | 'placebo'/de | 324 412 |
| #9 | 'clinical trial' OR 'clinical trials' | 1 217 560 |
| #10 | 'controlled clinical trial' OR 'controlled clinical trials' | 513 351 |
| #11 | 'randomised controlled trial' OR 'randomized controlled trial' OR 'randomised controlled trials' OR 'randomized controlled trials' | 415 651 |
| #12 | 'randomisation' OR 'randomization' | 672 814 |
| #13 | rct | 15 752 |
| #14 | 'random allocation' OR 'random assignment' | 64 104 |
| #15 | 'randomly allocated' OR 'randomly assigned' | 6169 |
| #16 | 'allocated randomly' OR 'assigned randomly' | 1561 |
| #17 | (single OR double OR triple OR treble) NEAR/1 (blind* OR mask*) | 204 035 |
| #18 | allocated NEAR/2 random OR assigned NEAR/2 random | 1561 |
| #19 | 'prospective study'/de | 295 755 |
| #20 | OR/#1-19 | 6 064 800 |
| #21 | 'case study'/de | 69 717 |
| #22 | 'case report' | 1 932 418 |
| #23 | 'abstract report'/de | 89 615 |
| #24 | 'letter'/de | 894 339 |
| #25 | 'case study'/de OR 'case report' OR 'abstract report'/de OR 'letter'/de | 2 795 719 |
| #27 | #20 NOT #25 | 5 896 392 |
| #28 | 'multiple sclerosis'/exp | 85 241 |
| #29 | 'encephalomyelitis'/exp | 25 227 |
| #30 | 'demyelinating disease'/exp | 108 223 |
| #31 | 'myelooptic neuropathy'/exp | 3623 |
| #32 | 'multiple sclerosis' OR 'neuromyelitis optica' OR encephalomyelitis OR devic OR rrms OR spms OR rms | 114 102 |
| #33 | #28 OR #29 OR #30 OR #31 OR #32 | 141 207 |
| #34 | 'beta interferon'/exp OR 'beta1 interferon'/exp OR 'beta1a interferon'/exp OR 'interferon beta serine'/exp OR 'recombinant beta interferon'/exp OR interferon NEAR/2 beta OR avonex OR belerofon OR ifn NEAR/2 beta OR ifn?beta OR beta1* NEAR/2 interferon OR betaferon OR rebif OR betaseron OR extavia | 24 735 |
| #35 | 'glatiramer'/exp OR glatiramer OR copaxone OR co*polymer*1 OR 'copolymer 1' OR 'co polymer 1' OR 'cop 1' | 5123 |
| #36 | 'natalizumab'/de OR natalizumab OR tysabri OR antegren OR 'anti vla4' OR 'anti-vla 4' OR 'anti alpha4 integrin' OR an100226 OR 'an 100226' | 5297 |
| #37 | 'fingolimod'/de OR fingolimod OR fty720 OR 'fty 720' OR Gilen?a | 3809 |
| #38 | 'alemtuzumab'/de OR alemtuzumab OR campath OR mabcampath OR 'ldp 103' OR ldp103 OR lemtrada | 10 041 |
| #39 | 'teriflunomide'/de OR teriflunomide OR 'a 771726' OR 'a77 1726' OR a771726 OR 'hmr 1726' OR hmr1726 OR 'rs 61980' OR rs61980 OR 'su 0020' OR su0020 or aubagio | 1002 |
| #40 | 'bg 00012' OR bg00012 OR 'bg 12' OR 'brn 0774590' OR panaclar or tecfidera or dmf | 7264 |
| #41 | 'peginterferon beta1a'/exp OR 'pegylated interferon beta' OR bib or plegridy | 277 |
| #42 | 'laquinimod'/exp OR 'abr 215062' OR abr215062 OR 'saik ms' OR 'tv 5600' OR tv5600 | 484 |
| #43 | #34 OR #35 OR #36 OR #37 OR #38 OR #39 OR #40 OR #41 OR #42 | 49 284 |
| #44 | #27 AND #33 AND #43 AND [1960-2012]/py | - |
| #45 | #27 AND #33 AND #43 AND [7-5-2013]/sd NOT [21-3-2014]/sd | 973 |
| #46 | #27 AND #33 AND #42 AND [31-10-2011]/sd NOT [21-3-2014]/sd | 165 |
| #47 | #45 OR #46 | 1070 |

Table B. List of Outcomes and Type of Sensitivity Analyses Conducted

| Outcome | Type of sensitivity analysis | Study selection criteria |
| --- | --- | --- |
| Annualized relapse rate | - Study duration | - Exclusion of studies with duration less than 1 year and greater than 2 years |
|  | - Blinding status | - Exclusion of studies with partial or assessor blinding or with unclear blinding status |
|  | - Sample size | - Exclusion of studies with <50 patients per treatment arm |
| CDP3M | - Study duration | - Kaplan-Meier survival curve at 1 year |
|  | - Blinding status | - Exclusion of studies with partial or assessor blinding or with unclear blinding status |
| CDP6M | - Study duration | - Kaplan-Meier survival curve at 1 year |
|  | - Blinding status | - Exclusion of studies with partial or assessor blinding or with unclear blinding status |
|  | - Sample size | - Exclusion of studies with <50 patients per treatment arm |

Abbreviations: CDP3M, 3-month confirmed disability progression; CDP6M, 6-month confirmed disability progression

**Statistical Methods A.**

Defining as the number of relapses in treatment arm k of trial i during the trial follow-up period, is the exposure time in person-years and is the rate at which event occur in arm k of trial I [28]:

The parameter of interest (rate) at which event occur in each trial arm was modelled on the log scale:

Events rate were modelled in Poisson regression accounting for different follow up time.

CDP was analyzed as a time-to-event outcome with data incorporated in the network meta-analysis (NMA) using a binomial likelihood:

where, is the cumulative count of subjects who have experienced an event (disability progression) in treatment group k of study s; is the total number of subjects in treatment group k of the study s. is the cumulative probability of a subject having experienced an event (or progression) [64].

A log cumulative hazard for each treatment arm was subsequently derived from as:

The log cumulative hazard estimates were then included in a treatment effect model with a linear regression structure and estimated as sum of study specific ‘baseline’ term and a treatment effect coefficient :

where, represents treatment effect for the baseline treatment in study *s*. The fixed study level ‘baseline’ term is a nuisance parameter, included to ensure that the treatment effect estimates are informed by within trial differences between treatment arms and not by differences in baseline event rates.

Treatment effect model under a proportional hazard assumption, the coefficient, is equal to the log cumulative hazard ratio over time, t:

where, represents the hazard for the baseline treatment in study s.

Table C. Network Meta-Analysis Results Using Fixed Effect Models for Efficacy Outcomes for Peginterferon Beta-1a 125 µg Every 2 Weeks Versus Comparators

| Comparison | IFN beta-1a 30 µg QW | IFN beta-1a 22 µg TIW | IFN beta-1a 44 µg TIW | IFN beta-1b 250 µg EOD | GA 20 mg OD | Placebo |
| --- | --- | --- | --- | --- | --- | --- |
| ARR | 0.870 (0.676 – 1.129) | 0.913 (0.694 – 1.201) | 0.984 (0.762 – 1.279) | 0.949 (0.727 – 1.234) | 1.002 (0.779 – 1.293) | 0.651 (0.513 – 0.823) |
| CDP3M | 0.765 (0.439 – 1.316) | 0.808 (0.457 – 1.407) | 0.908 (0.526 – 1.518) | 0.726 (0.423 – 1.223) | 0.718 (0.422 – 1.193) | 0.588 (0.372 – 0.910) |
| CDP6M | 0.551 (0.280 – 1.037) | - | 0.559 (0.268 – 1.148) | 0.924 (0.204 – 4.653) | 0.619 (0.306 – 1.232) | 0.434 (0.241 – 0.744) |

Results presented as Effect Size (95% CrI); Effect size <1 indicates favorable efficacy of intervention for ARR and CDP outcomes, <0 for change outcomes; Rate ratios presented for ARR, Hazard Ratios for disability outcomes, Risk ratio for relapse free outcome, and absolute difference for continuous outcomes; Highlighted cells represents statistically significant results

Abbreviations: ARR: Annualized relapse Rate; CDP3M: 3-month confirmed disability progression; CDP6M: 6-month confirmed disability progression; CrI: Credible Interval; EOD: Every Other Day; IFN: interferon; OD: once daily; PEG: Pegylated; QW: once weekly; TIW: 3 times a week

Table D. Comparison of Model Complexities Between Random Effect and Fixed Effect Models

| Outcome | Analysis type | Residual deviance | | Tau^2^ value | | DIC | pD |
| --- | --- | --- | --- | --- | --- | --- | --- |
|  |  | Mean (SD) | Median (95% CrI) | Mean (SD) | Median (95% CrI) |  |  |
| ARR | Random effect | 38.57 (7.32) | 38.03 (26.05 – 54.29) | 0.006115 (0.01088) | 0.002325 (0.00002540 – 0.03508) | 293.962 | 24.651 |
|  | Fixed effect | 39.39 (6.66) | 38.71 (28.40 – 54.17) | - | - | 292.070 | 21.988 |
| CDP3M | Random effect | - | - | 0.2292 (0.1986) | 0.1777 (0.06245 – 0.7085) | 123.140 | 13.517 |
|  | Fixed effect | - | - | - | - | 123.410 | 13.933 |
| CDP6M | Random effect | - | - | 0.3007 (0.3339) | 0.2167 (0.06478 – 1.047) | 96.177 | 11.049 |
|  | Fixed effect | - | - | - | - | 97.311 | 11.949 |

Abbreviations: ARR, annualized relapse rate; CDP3M: 3-month confirmed disability progression; CDP6M: 6-month confirmed disability progression; CrI, credible interval; DIC, deviance information criteria; pD, effective number of parameters; SD, standard deviation; lower DIC indicates a good model fit

Table E. Model Fit Statistics With and Without INCOMIN Trial Data

| **Model fit with INCOMIN** | | | **Model fit without INCOMIN** | |
| --- | --- | --- | --- | --- |
| **Model** | **Fixed Effect Model** | **Random Effects Model** | **Fixed Effect Model** | **Random Effects Model** |
| **Dbar** | 17.93* | 17.93* | 16.64^#^ | 16.34^#^ |
| **pD** | 13.17 | 12.35 | 12.12 | 11.20 |
| **DIC** | 31.10 | 30.28 | 28.76 | 27.54 |
| **Tau^2^** |  | 0.2852 |  | 0.2167 |

Abbreviations: Dbar, mean difference; DIC, deviance information criteria; pD, effective number of parameters; lower DIC indicates a good model fit

*Compare to 16 data points; #compare to 14 data points.

Table F. Risk of Bias Assessment for Included RCTs

| Trial | Randomization | Baseline Characteristics | Blinding | Withdrawal/ Discontinuation | Outcome Selection, Reporting, and Other Sources  of Bias | Statistical Analysis |
| --- | --- | --- | --- | --- | --- | --- |
| ADVANCE trial[24] | LR | LR | LR | LR | LR | LR |
| BECOME trial[49] | NR | LR | LR | LR | LR | LR |
| BEYOND trial[48] | LR | LR | LR | LR | LR | LR |
| Bornstein 1987[51] | NR | LR | LR | NR | NR | LR |
| BRAVO trial[63] | LR | LR | LR | LR | LR | LR |
| Calabrese 2011[41] | LR | NR | NR | LR | NR | HR |
| CombiRx trial[46] | LR | LR | LR | LR | LR | LR |
| CONFIRM trial*[47] | LR | LR | LR | LR | LR | LR |
| Copolymer 1  MS trial[52] | NR | LR | LR | LR | NR | LR |
| Etemadifar 2006[45] | NR | LR | LR | LR | NR | LR |
| European and Canadian  glatiramer trial[53] | LR | LR | LR | LR | NR | LR |
| EVIDENCE trial[44] | LR | LR | LR | LR | NR | LR |
| IFNB MS trial[42] | NR | LR | LR | LR | NR | LR |
| MSCRG Trial[50] | LR | LR | LR | NR | NR | LR |
| PRISMS trial[43] | LR | LR | LR | LR | NR | LR |
| REGARD trial[54] | LR | LR | LR | LR | LR | LR |

Abbreviations: HR, high risk of bias; LR, low risk of bias; NR, not reported

*Bias assessment was performed on the basis of overall study design (double-blind); however, the active comparator was open-label and rater-masked in the CONFIRM trial.

Randomization: Was randomization carried out appropriately? Concealment grade: Was the concealment of treatment allocation adequate?

Blinding: Were the care providers, participants, and outcome assessors blind to treatment allocation?

Baseline comparability: Were the groups similar at the outset of the study in terms of prognostic factors?

Follow-up: Were there any unexpected imbalances in drop-outs between groups? Selective reporting and other sources of bias: Is there any evidence to suggest that the authors measured more outcomes than they reported? State any important concerns about bias not addressed in the other domains in the tool. If particular questions/entries were pre-specified in the review’s protocol, responses should be provided for each question/entry.

Analysis: Did the analysis include an intention-to-treat analysis? If so, was this appropriate and were appropriate methods used to account for missing data?

Fig A. Percentage of Studies Presenting a Risk of Bias

*
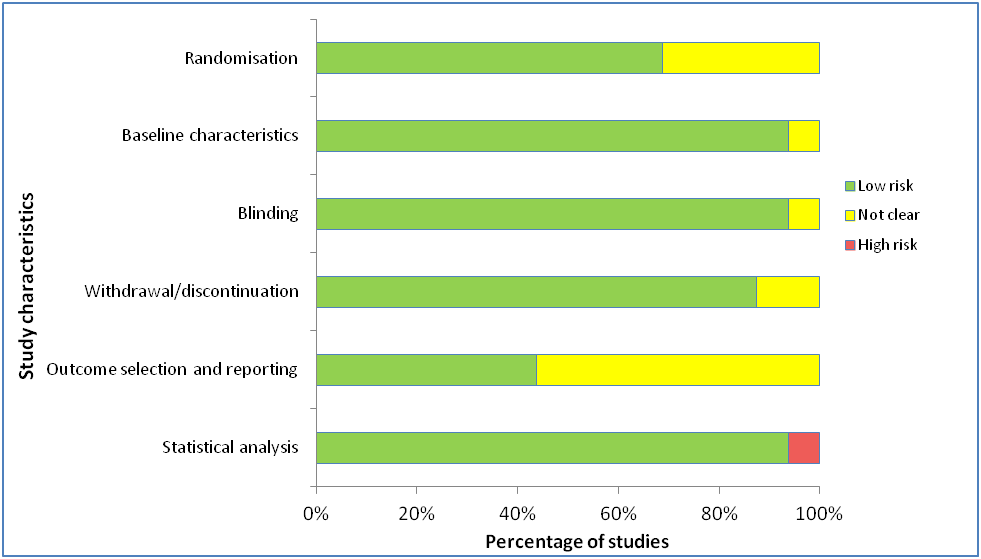
*

Fig B. Jadad Score (A) and Treatment Allocation Concealment (B) in the Included Studies; Maximum Jadad Score = 5

(1)


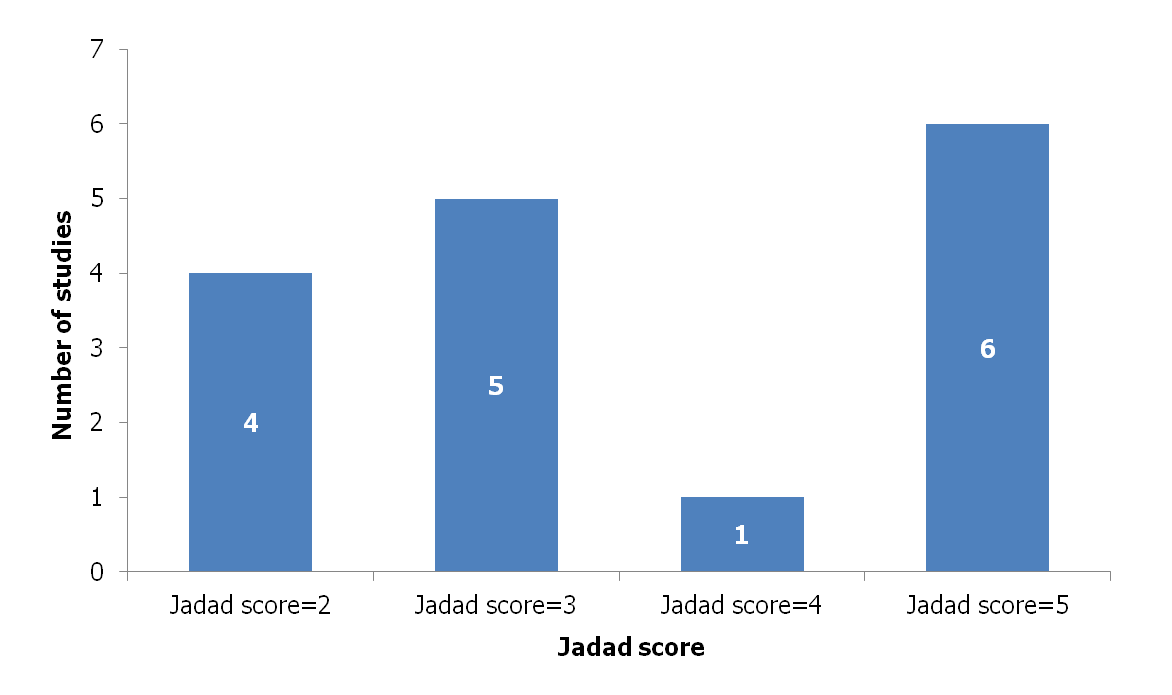


(2)

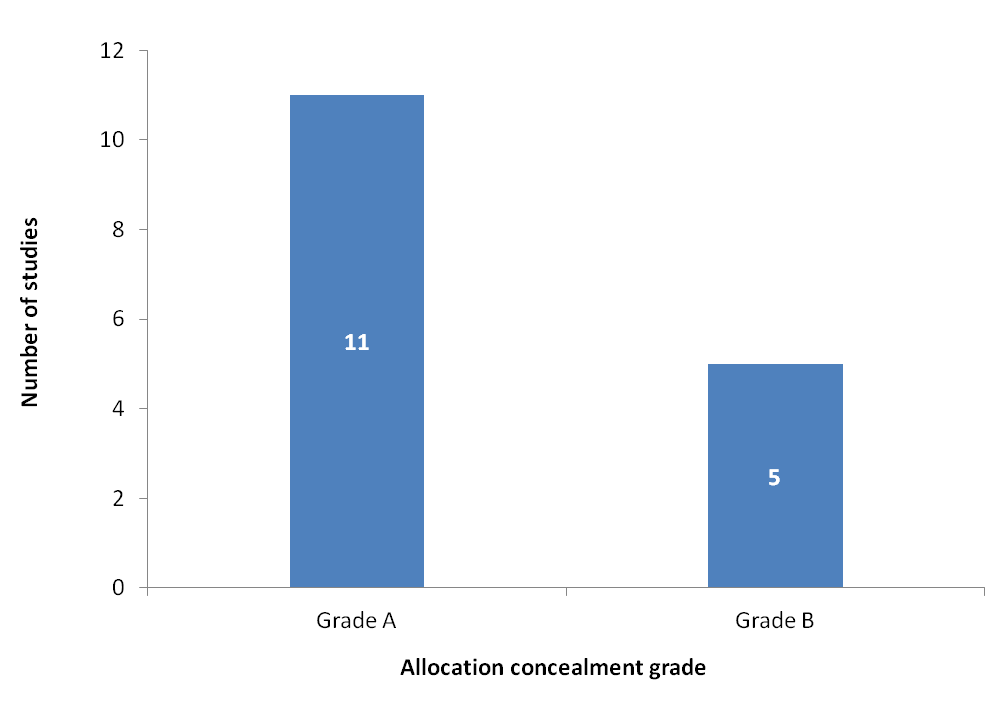


Table G. 3-month confirmed disability progression data used in the network meta-analysis

| Study Name | Time point | Treatments | ITT N | Patients with disease progression (n) |
| --- | --- | --- | --- | --- |
| ADVANCE trial | 12 months | Placebo | 500 | 50 |
| ADVANCE trial | 12 months | PEG-IFN beta-1a 125 µg every 2 weeks | 515 | 31 |
| BEYOND trial | 24 months | GA 20 mg OD | 448 | 90 |
| BEYOND trial | 24 months | IFN beta-1b 250 µg EOD | 897 | 188 |
| CONFIRM trial | 24 months | GA 20 mg OD | 360 | 48 |
| CONFIRM trial | 24 months | Placebo | 363 | 52 |
| Copolymer 1 trial | 24 months | GA 20 mg OD | 125 | 27 |
| Copolymer 1 trial | 24 months | Placebo | 126 | 31 |
| EVIDENCE trial | 12 months | IFN beta-1a 30 µg once weekly | 338 | 49 |
| EVIDENCE trial | 12 months | IFN beta-1a 44 µg TIW | 339 | 43 |
| IFNB MS trial | 60 months | IFN beta-1b 250 µg EOD | 124 | 43 |
| IFNB MS trial | 60 months | Placebo | 123 | 56 |
| PRISMS trial | 24 months | IFN beta-1a 22 µg TIW | 189 | 56 |
| PRISMS trial | 24 months | IFN beta-1a 44 µg TIW | 184 | 49 |
| PRISMS trial | 24 months | Placebo | 187 | 72 |
| BRAVO trial | 24 months | Placebo | 450 | 60 |
| BRAVO trial | 24 months | IFN beta-1a, 30 µg, once weekly | 447 | 47 |

Table H. 6-month confirmed disability progression data used in the network meta-analysis

| Study Name | Time point | Treatments | ITT N | No. with progression (n) |
| --- | --- | --- | --- | --- |
| ADVANCE trial | 12 months | Placebo | 500 | 39 |
| ADVANCE trial | 12 months | PEG-IFN beta-1a 125 µg every 2 weeks | 515 | 18 |
| BECOME trial | 24 months | GA 20 mg OD | 39 | 6 |
| BECOME trial | 24 months | IFN beta-1b 250 µg EOD | 36 | 4 |
| CONFIRM trial | 24 months | GA 20 mg OD | 360 | 34 |
| CONFIRM trial | 24 months | Placebo | 363 | 39 |
| EVIDENCE trial | 12 months | IFN beta-1a 30 µg once weekly | 338 | 28 |
| EVIDENCE trial | 12 months | IFN beta-1a 44 µg TIW | 339 | 20 |
| MSCRG Trial | 12 months | IFN beta-1a 30 µg once weekly | 158 | 20 |
| MSCRG Trial | 12 months | Placebo | 143 | 31 |
| REGARD trial | 24 months | GA 20 mg OD | 378 | 33 |
| REGARD trial | 24 months | IFN beta-1a 44 µg TIW | 386 | 45 |
| BRAVO trial | 24 months | Placebo | 450 | 46 |
| BRAVO trial | 24 months | IFN beta-1a, 30 µg, once weekly | 447 | 35 |

Table I. Annualized relapse rate data used in the network meta-analysis

| Study Name | Treatments | Person Years | number of relapses |
| --- | --- | --- | --- |
| BECOME trial | GA 20 mg OD | 69.7 | 23 |
| BECOME trial | IFN beta-1b 250 µg EOD | 67.57 | 25 |
| BEYOND trial | GA 20 mg OD | 1099.5 | 374 |
| BEYOND trial | IFN beta-1b 250 µg EOD | 2260 | 814 |
| Bornstein 1987 | GA 20 mg OD | 23.67 | 8 |
| Bornstein 1987 | Placebo | 27.59 | 38 |
| Calabrese 2011 | GA 20 mg OD | 103 | 52 |
| Calabrese 2011 | IFN beta-1a 30 µg once weekly | 102 | 51 |
| Calabrese 2011 | IFN beta-1a 44 µg TIW | 101 | 40 |
| CONFIRM trial | GA 20 mg OD | 300.91 | 105 |
| CONFIRM trial | Placebo | 312.62 | 146 |
| Copolymer 1 trial | GA 20 mg OD | 272.88 | 161 |
| Copolymer 1 trial | Placebo | 250 | 210 |
| Etemadafir 2006 | IFN beta-1a 30 µg once weekly | 60 | 57 |
| Etemadafir 2006 | IFN beta-1a 44 µg TIW | 60 | 66 |
| Etemadafir 2006 | IFN beta-1b 250 µg EOD | 60 | 65 |
| European/Canadian GA trial | GA 20 mg OD | 75.31 | 61 |
| European/Canadian GA trial | Placebo | 75.21 | 91 |
| EVIDENCE trial | IFN beta-1a 30 µg once weekly | 304.2 | 195 |
| EVIDENCE trial | IFN beta-1a 44 µg TIW | 304.71 | 165 |
| IFNB-MS trial | IFN beta-1b 250 µg EOD | 207 | 173 |
| IFNB-MS trial | Placebo | 209.2 | 266 |
| MSCRG trial | IFN beta-1a 30 µg once weekly | 170 | 104 |
| MSCRG trial | Placebo | 174 | 157 |
| PRISMS trial | IFN b-1a 22 µg TIW | 366 | 344 |
| PRISMS trial | IFN beta-1a 44 µg TIW | 363 | 318 |
| PRISMS trial | Placebo | 364 | 479 |
| REGARD trial | GA 20 mg OD | 669.5 | 194 |
| REGARD trial | IFN beta-1a 44 µg TIW | 669.5 | 201 |
| ADVANCE trial | Placebo | 445.25 | 181 |
| ADVANCE trial | PEG-IFN beta-1a 125 µg every 2 weeks | 435.74 | 116 |
| CombiRx trial | IFN beta-1a 30 µg once weekly | 604.4 | 97 |
| CombiRx trial | GA 20 mg OD | 650.7 | 70 |
| BRAVO trial | IFN beta-1a 30 µg once weekly | 825 | 215 |
| BRAVO trial | Placebo | 809 | 275 |

Table J. Results from the network meta-analysis comparing the treatments (treatments in rows vs. treatments in columns) for annualized relapse rate. The Rate Ratios and corresponding 95% confidence intervals are provided

|  | Placebo | IFN beta-1a 30 µg once weekly | IFN beta-1b 250 µg EOD | IFN b-1a 22 µg TIW | IFN beta-1a 44 µg TIW | GA 20 mg OD | PEG-IFN beta-1a 125 µg every 2 weeks |
| --- | --- | --- | --- | --- | --- | --- | --- |
| Placebo | - | 1.34 (1.19 to 1.54) | 1.46 (1.25 to 1.71) | 1.41 (1.16 to 1.71) | 1.51 (1.33 to 1.75) | 1.54 (1.37 to 1.75) | 1.54 (1.15 to 2.03) |
| IFN beta-1a 30 µg once weekly | 0.74 (0.65 to 0.84) | - | 1.08 (0.91 to 1.31) | 1.05 (0.83 to 1.3) | 1.12 (0.98 to 1.3) | 1.15 (0.99 to 1.33) | 1.14 (0.84 to 1.54) |
| IFN beta-1b 250 µg EOD | 0.68 (0.58 to 0.8) | 0.92 (0.76 to 1.1) | - | 0.96 (0.75 to 1.22) | 1.04 (0.86 to 1.23) | 1.05 (0.92 to 1.23) | 1.05 (0.77 to 1.44) |
| IFN b-1a 22 µg TIW | 0.71 (0.59 to 0.86) | 0.96 (0.77 to 1.2) | 1.04 (0.82 to 1.33) | - | 1.07 (0.88 to 1.32) | 1.09 (0.89 to 1.37) | 1.09 (0.77 to 1.56) |
| IFN beta-1a 44 µg TIW | 0.66 (0.57 to 0.75) | 0.89 (0.77 to 1.02) | 0.97 (0.81 to 1.16) | 0.93 (0.76 to 1.13) | - | 1.02 (0.89 to 1.18) | 1.02 (0.74 to 1.38) |
| GA 20 mg OD | 0.65 (0.57 to 0.73) | 0.87 (0.75 to 1.01) | 0.95 (0.82 to 1.08) | 0.91 (0.73 to 1.13) | 0.98 (0.85 to 1.13) | - | 1 (0.73 to 1.34) |
| PEG-IFN beta-1a 125 µg every 2 weeks | 0.65 (0.49 to 0.87) | 0.88 (0.65 to 1.2) | 0.95 (0.7 to 1.31) | 0.92 (0.64 to 1.3) | 0.98 (0.72 to 1.36) | 1 (0.75 to 1.37) | - |

Table K. Results from the network meta-analysis comparing the treatments (treatments in rows vs. treatments in columns) for 3-month confirmed disability progression. The Hazard Ratios and corresponding 95% confidence intervals are provided

|  | Placebo | IFN beta-1a 30 µg once weekly | IFN beta-1b 250 µg EOD | IFN beta-1a 22 µg TIW | IFN beta-1a 44 µg TIW | GA 20 mg OD | PEG-IFN beta-1a µg every 2 weeks |
| --- | --- | --- | --- | --- | --- | --- | --- |
| Placebo | - | 1.27 (0.93 to 1.73) | 1.23 (0.93 to 1.61) | 1.29 (0.93 to 1.83) | 1.44 (1.07 to 1.96) | 1.22 (0.95 to 1.58) | 1.73 (1.12 to 2.73) |
| IFN beta-1a 30 µg once weekly | 0.78 (0.58 to 1.07) | - | 0.96 (0.65 to 1.44) | 1.02 (0.67 to 1.54) | 1.13 (0.82 to 1.59) | 0.96 (0.65 to 1.41) | 1.36 (0.8 to 2.34) |
| IFN beta-1b 250 µg EOD | 0.82 (0.62 to 1.07) | 1.04 (0.7 to 1.53) | - | 1.06 (0.69 to 1.63) | 1.18 (0.79 to 1.75) | 1 (0.8 to 1.25) | 1.41 (0.85 to 2.39) |
| IFN beta-1a 22 µg TIW | 0.77 (0.55 to 1.08) | 0.98 (0.65 to 1.49) | 0.95 (0.61 to 1.45) | - | 1.12 (0.77 to 1.61) | 0.94 (0.62 to 1.44) | 1.34 (0.77 to 2.38) |
| IFN beta-1a 44 µg TIW | 0.69 (0.51 to 0.94) | 0.88 (0.63 to 1.22) | 0.85 (0.57 to 1.27) | 0.9 (0.62 to 1.29) | - | 0.85 (0.57 to 1.25) | 1.2 (0.7 to 2.07) |
| GA 20 mg OD | 0.82 (0.63 to 1.05) | 1.04 (0.71 to 1.53) | 1 (0.8 to 1.25) | 1.06 (0.7 to 1.63) | 1.18 (0.8 to 1.74) | - | 1.42 (0.86 to 2.38) |
| PEG-IFN beta-1a µg every 2 weeks | 0.58 (0.37 to 0.89) | 0.74 (0.43 to 1.26) | 0.71 (0.42 to 1.17) | 0.75 (0.42 to 1.3) | 0.83 (0.48 to 1.43) | 0.71 (0.42 to 1.16) | - |

Table L. Results from the network meta-analysis comparing the treatments (treatments in rows vs. treatments in columns) for 6-month confirmed disability progression. The Hazard Ratios and corresponding 95% confidence intervals are provided

|  | Placebo | IFN beta-1a 30 µg once weekly | IFN beta-1b 250 µg EOD | IFN beta-1a 44 µg TIW | GA 20 mg OD | PEG-IFN beta-1a 125 µg every 2 weeks |
| --- | --- | --- | --- | --- | --- | --- |
| Placebo | - | 1.25 (0.91 to 1.71) | 1.87 (0.59 to 7.16) | 1.29 (0.83 to 1.97) | 1.44 (0.97 to 2.07) | 2.32 (1.37 to 4.12) |
| IFN beta-1a 30 µg once weekly | 0.8 (0.59 to 1.1) | - | 1.5 (0.47 to 5.86) | 1.03 (0.67 to 1.58) | 1.16 (0.75 to 1.77) | 1.87 (1.01 to 3.56) |
| IFN beta-1b 250 µg EOD | 0.53 (0.14 to 1.7) | 0.66 (0.17 to 2.13) | - | 0.69 (0.18 to 2.23) | 0.77 (0.21 to 2.39) | 1.25 (0.3 to 4.38) |
| IFN beta-1a 44 µg TIW | 0.78 (0.51 to 1.21) | 0.97 (0.64 to 1.5) | 1.46 (0.45 to 5.7) |  | 1.12 (0.76 to 1.64) | 1.81 (0.92 to 3.63) |
| GA 20 mg OD | 0.69 (0.48 to 1.03) | 0.86 (0.57 to 1.33) | 1.3 (0.42 to 4.85) | 0.9 (0.61 to 1.31) | - | 1.62 (0.84 to 3.18) |
| PEG-IFN beta-1a µg every 2 weeks | 0.43 (0.24 to 0.73) | 0.54 (0.28 to 0.99) | 0.8 (0.23 to 3.31) | 0.55 (0.28 to 1.09) | 0.62 (0.31 to 1.19) | - |

Fig C. Annualised Relapse Rate Across Studies for the Placebo Group


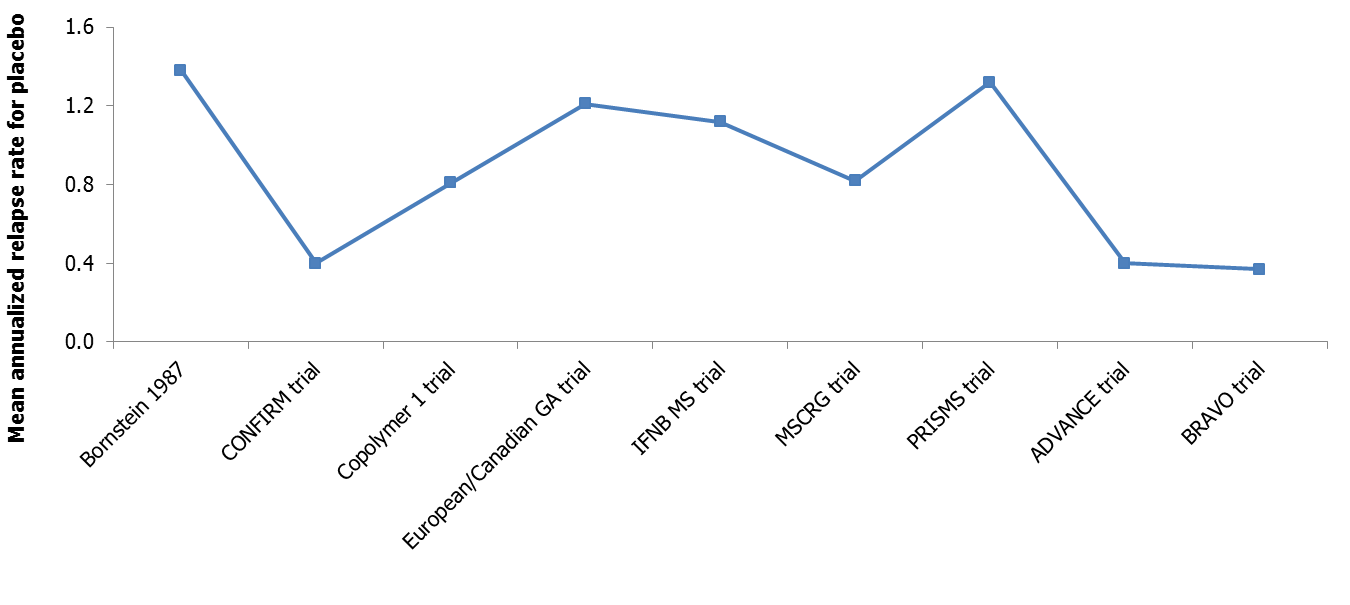


Table M. Probability of Treatment Being Ranked 1 – 7 for Best ARR Outcome (Standard Deviations are in Parentheses)

|  | Rank 1 | Rank 2 | Rank 3 | Rank 4 | Rank 5 | Rank 6 | Rank 7 | SUCRA |
| --- | --- | --- | --- | --- | --- | --- | --- | --- |
| Placebo | 0 (0) | 0 (0) | 0 (0) | 0 (0) | 0 (0.01) | 0.01 (0.07) | 0.99 (0.08) | 0.00 |
| IFN beta-1a 30 µg QW | 0 (0.06) | 0.02 (0.13) | 0.05 (0.21) | 0.14 (0.35) | 0.31 (0.46) | 0.49 (0.5) | 0 (0.01) | 0.30 |
| IFN beta-1b 250 µg EOD | 0.07 (0.25) | 0.15 (0.36) | 0.23 (0.42) | 0.27 (0.44) | 0.2 (0.4) | 0.08 (0.27) | 0 (0.01) | 0.56 |
| IFN b-1a 22 µg TIW | 0.07 (0.25) | 0.09 (0.29) | 0.13 (0.33) | 0.19 (0.39) | 0.28 (0.45) | 0.25 (0.43) | 0 (0.05) | 0.46 |
| IFN beta-1a 44 µg TIW | 0.18 (0.38) | 0.27 (0.44) | 0.26 (0.44) | 0.21 (0.41) | 0.08 (0.26) | 0.01 (0.1) | 0 (0.01) | 0.71 |
| GA 20 mg OD | 0.26 (0.44) | 0.36 (0.48) | 0.25 (0.43) | 0.1 (0.3) | 0.02 (0.15) | 0.01 (0.08) | 0 (0) | 0.79 |
| PEG-IFN beta-1a 125 µg every 2 weeks | 0.42 (0.49) | 0.11 (0.31) | 0.09 (0.29) | 0.1 (0.3) | 0.11 (0.31) | 0.16 (0.37) | 0 (0.06) | 0.69 |

Table N. Probability of Treatment Being Ranked 1-7 for Best CDP3M Outcome (Standard Deviations are in Parentheses)

|  | Rank 1 | Rank 2 | Rank 3 | Rank 4 | Rank 5 | Rank 6 | Rank 7 | SUCRA |
| --- | --- | --- | --- | --- | --- | --- | --- | --- |
| Placebo | 0 (0) | 0 (0.01) | 0 (0.03) | 0 (0.07) | 0.04 (0.2) | 0.18 (0.38) | 0.78 (0.42) | 0.05 |
| IFN beta-1a 30 µg QW | 0.04 (0.2) | 0.14 (0.34) | 0.21 (0.41) | 0.22 (0.41) | 0.17 (0.37) | 0.18 (0.38) | 0.05 (0.22) | 0.49 |
| IFN beta-1b 250 µg EOD | 0.02 (0.15) | 0.1 (0.3) | 0.14 (0.35) | 0.21 (0.41) | 0.27 (0.44) | 0.21 (0.4) | 0.06 (0.23) | 0.43 |
| IFN beta-1a 22 µg TIW | 0.07 (0.25) | 0.18 (0.38) | 0.2 (0.4) | 0.19 (0.39) | 0.14 (0.35) | 0.17 (0.37) | 0.06 (0.23) | 0.52 |
| IFN beta-1a 44 µg TIW | 0.18 (0.38) | 0.37 (0.48) | 0.22 (0.41) | 0.12 (0.33) | 0.08 (0.27) | 0.03 (0.18) | 0 (0.06) | 0.72 |
| GA 20 mg OD | 0.02 (0.13) | 0.08 (0.28) | 0.15 (0.36) | 0.21 (0.41) | 0.28 (0.45) | 0.22 (0.41) | 0.05 (0.21) | 0.42 |
| PEG-IFN beta-1a 125 µg every 2 weeks | 0.67 (0.47) | 0.14 (0.34) | 0.08 (0.28) | 0.05 (0.22) | 0.03 (0.17) | 0.02 (0.15) | 0 (0.07) | 0.88 |

Fig D. Estimated Proportion of Patients with 3-Month Confirmed Disability Progression Across Studies for the Placebo Group


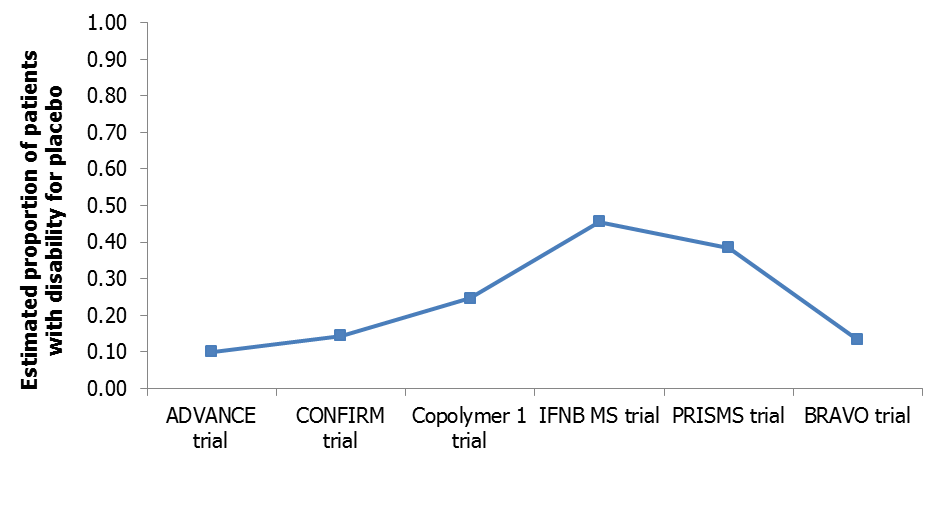


Fig E. Estimated Proportion of Patients with 6-Month Confirmed Disability Progression Across Studies for the Placebo Group


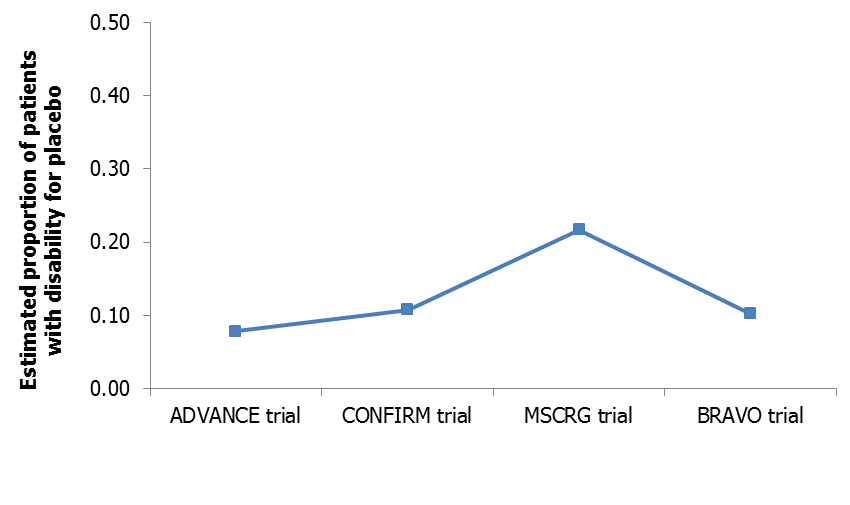


Table O. Probability of Treatment Being Ranked 1-7 for Best CDP6M Outcome (Standard Deviations are in Parentheses)

|  | Rank 1 | Rank 2 | Rank 3 | Rank 4 | Rank 5 | Rank 6 | SUCRA |
| --- | --- | --- | --- | --- | --- | --- | --- |
| Placebo | 0 (0) | 0 (0.03) | 0.01 (0.09) | 0.04 (0.21) | 0.21 (0.41) | 0.74 (0.44) | 0.1 |
| IFN beta-1a 30 µg QW | 0 (0.07) | 0.08 (0.27) | 0.2 (0.4) | 0.31 (0.46) | 0.36 (0.48) | 0.04 (0.2) | 0.4 |
| IFN beta-1b 250 µg EOD | 0.38 (0.48) | 0.26 (0.44) | 0.08 (0.27) | 0.07 (0.25) | 0.08 (0.28) | 0.13 (0.34) | 0.7 |
| IFN beta-1a 44 µg TIW | 0.01 (0.12) | 0.1 (0.3) | 0.23 (0.42) | 0.33 (0.47) | 0.24 (0.43) | 0.08 (0.27) | 0.4 |
| GA 20 mg OD | 0.02 (0.15) | 0.22 (0.42) | 0.43 (0.49) | 0.23 (0.42) | 0.09 (0.29) | 0.01 (0.09) | 0.6 |
| PEG-IFN beta-1a 125 µg every 2 weeks | 0.58 (0.49) | 0.34 (0.47) | 0.05 (0.22) | 0.02 (0.15) | 0.01 (0.1) | 0 (0.02) | 0.9 |

Table P. Summary of Sensitivity Analysis Between Peginterferon Beta-1a and Comparators for Annualized Relapse Rate

| Comparator | Annualized Relapse Rate (95% CrI) | | | |
| --- | --- | --- | --- | --- |
|  | Overall Results | Blinding Sensitivity | Sample Size Sensitivity | Study Duration Sensitivity |
| Peginterferon beta-1a 125 µg every 2 weeks vs | | | | |
| IFN beta-1a 30 µg QW | 0.877  (0.650–1.196) | 0.860  (0.239–3.408) | 0.864  (0.632–1.141) | 0.900  (0.675–1.237) |
| IFN beta-1b 250 µg EOD | 0.954  (0.696–1.307) | 1.035  (0.295–4.213) | 0.986  (0.688–1.311) | 0.932  (0.696–1.286) |
| IFN beta-1a 22 µg TIW | 0.916  (0.642–1.300) | 0.911  (0.200–4.111) | 0.956  (0.660–1.301) | 0.911  (0.663–1.289) |
| IFN beta-1a 44 µg TIW | 0.984  (0.724–1.359) | 0.974  (0.213–4.451) | 1.022  (0.737–1.365) | 0.983  (0.735–1.364) |
| GA 20 mg OD | 1.001  (0.749–1.373) | 1.143  (0.378–4.237) | 1.019 (0.735–1.321) | 0.977  (0.736–1.343) |
| Placebo | 0.650  (0.493–0.867) | 0.650  (0.227–1.873) | 0.670  (0.501–0.863) | 0.649  (0.502–0.860) |
| Tau^2^ value (median, 95% CrI) | 0.002325 (0.0000254, 0.03508) | 0.0924  (0.0006355, 1.747) | 0.001578 (0.0000001399, 0.03087) | 0.002091 (0.000009196, 0.03333) |
| DIC | 293.962 | 168.461 | 240.18 | 261.803 |
| PD | 24.651 | 18.507 | 20.997 | 22.175 |

Abbreviations: CrI, credible interval; DIC, deviance information criteria; EOD, every other day; GA, glatiramer acetate; IFN, interferon; µg, microgram; mg, milligram; OD, once daily; PD, posterior deviance; PEG, pegylated; QW, once weekly; TIW, 3 times a week.

Highlighted cells represent statistically significant results.

Table Q. Summary of Sensitivity Analysis Between Peginterferon Beta-1a and Comparators for 3-month Confirmed Disability Progression

| Comparator | 3-month Confirmed Disability Progression (95% CrI) | | |
| --- | --- | --- | --- |
|  | Overall Results | Blinding Sensitivity | Kaplan-Meier Sensitivity |
| Peginterferon beta-1a 125 µg every 2 weeks vs. | | | |
| IFN beta-1a 30 µg QW | 0.737 (0.427–1.257) | - | 0.774 (0.444–1.37) |
| IFN beta-1b 250 µg EOD | 0.71 (0.419–1.172) | 0.731 (0.427–1.213) | 0.642 (0.286–1.466) |
| IFN beta-1a 22 µg TIW | 0.749 (0.421–1.295) | 0.707 (0.391–1.253) | 0.978 (0.534–1.825) |
| IFN beta-1a 44 µg TIW | 0.834 (0.485–1.425) | 0.804 (0.441–1.446) | 0.878 (0.507–1.543) |
| GA 20 mg OD | 0.707 (0.42–1.159) | 0.738 (0.427–1.249) | 0.559 (0.296–1.043) |
| Placebo | 0.579 (0.36–0.891) | 0.549 (0.345–0.848) | 0.577 (0.368–0.889) |
| Tau^2^ value (median, 95% CrI) | 0.1777 (0.06245, 0.7085) | 0.1003 (0.01783, 0.8361) | 0.2876 (0.08495, 1.592) |
| DIC | 123.14 | 91.417 | 88.43 |
| PD | 13.517 | 11.58 | 10.655 |

Abbreviations: CrI, credible interval; DIC, deviance information criteria; EOD, every other day; GA, glatiramer acetate; IFN, interferon; OD, once daily; PD, posterior deviance; PEG, pegylated; QW, once weekly; TIW, 3 times a week.

Highlighted cells represent statistically significant results.

Table R. Summary of Sensitivity Analysis Between Peginterferon Beta-1a and Comparators for 6-month Confirmed Disability Progression

| Comparator | 6-month Confirmed Disability Progression (95% CrI) | | | | |
| --- | --- | --- | --- | --- | --- |
|  | Overall Results | | Blinding Sensitivity | Sample Size Sensitivity | Kaplan-Meier Sensitivity |
| Peginterferon beta-1a 125 µg every 2 weeks vs. | | | | | |
| IFN beta-1a 30 µg QW | 0.535 (0.282–0.987) | 0.740  (0.326–1.652) | | 0.553  (0.292–1.021) | 0.426  (0.205–0.866) |
| IFN beta-1b 250 µg EOD | 0.799 (0.229–3.313) | - | | - | - |
| IFN beta-1a 44 µg TIW | 0.553 (0.275–1.091) | - | | 0.576  (0.280–1.151) | 0.926  (0.368–2.359) |
| GA 20 mg OD | 0.619 (0.315–1.186) | - | | 0.643  (0.321–1.235) | 0.392  (0.214–0.689) |
| Placebo | 0.431 (0.243–0.732) | 0.422  (0.235–0.721) | | 0.433  (0.246–0.740) | 0.418  (0.236–0.701) |
| Tau^2^ value (median, 95% CrI) | 0.2167 (0.06478, 1.047) | 1.113 (0.0734, 380.7) | | 0.2463 (0.06962, 1.373) | 0.2793 (0.0399, 3.497) |
| DIC | 96.177 | 28.066 | | 86.305 | 56.205 |
| PD | 11.049 | 4.038 | | 9.594 | 7.808 |

Abbreviations: CrI, credible interval; DIC, deviance information criteria; EOD, every other day; GA, glatiramer acetate; IFN, interferon; OD, once daily; PD, posterior deviance; PEG, pegylated; QW, once weekly; TIW, 3 times a week.

Highlighted cells represent statistically significant results.
